# Dual targeting of the mitochondrial Lon peptidase 1 and the chymotrypsin-like proteasome activity as a potential therapeutic strategy in malignant astrocytoma models

**DOI:** 10.1016/j.phrs.2025.107697

**Published:** 2025-03-13

**Authors:** Christopher Douglas, Shashi Jain, Naomi Lomeli, Javier Lepe, Kaijun Di, Nitesh Kumar Nandwana, Adil Shareef Mohammed, Thao Vu, James Pham, Maria Cristina Kenney, Bhaskar Das, Daniela A. Bota

**Affiliations:** a Department of Experimental Pathology & Laboratory Medicine, University of California Irvine, Irvine, CA, USA; b Department of Neurology, University of California Irvine, Irvine, CA, USA; c Department of Ophthalmology Research, University of California Irvine, Irvine, CA, USA; d University at Buffalo, The State University of New York (SUNY), USA; e School of Pharmacy and Pharmaceutical Sciences, SUNY, NY, USA; f Chao Family Comprehensive Cancer Center, University of California Irvine, Irvine, CA, USA

**Keywords:** Malignant astrocytoma, Glioblastoma, LonP1, CT-L proteasome, BT317, reactive oxygen species

## Abstract

Malignant astrocytomas are aggressive primary brain tumors characterized by extensive hypoxia-induced, mitochondria-dependent changes such as altered respiration, increased chymotrypsin-like (CT-L) proteasome activity, decreased apoptosis, drug resistance, stemness, and increased invasiveness. Mitochondrial Lon Peptidase 1 (LonP1) overexpression and increased CT-L proteasome activity are biomarkers of an aggressive high-grade phenotype and found to be associated with recurrence and poor patient survival. In preclinical models, small molecule agents targeting either LonP1 or the proteasome CT-L activity have anti-astrocytoma activity. Here, we present evidence that the dual inhibition of LonP1 and CT-L proteasome activity effectively induces ROS production, leading to apoptosis in malignant astrocytoma established cell lines and patient-derived glioma stem cell-like cultures. We also evaluated a novel small molecule, BT317, derived from the coumarinic compound 4 (CC4) using structure-activity modeling, which we found to inhibit both LonP1 and CT-L proteasome activity. Using gain- and loss-of-function genetic models, we discovered that LonP1 is both necessary and sufficient to drive BT317 drug sensitivity in established and patient-derived glioma stem-like cells by generating ROS and inducing apoptosis. *In vitro*, BT317 had activity as a single agent but, more importantly, enhanced synergy with the standard of care commonly used chemotherapeutic temozolomide (TMZ). In an orthotopic xenograft astrocytoma model, BT317 crossed the blood-brain barrier, showed selective activity at the tumor site, and demonstrated therapeutic efficacy as a single agent and combined with TMZ. BT317 defines an emerging class of LonP1 and CT-L inhibitors that exhibited promising anti-tumor activity and could be a potential candidate for malignant astrocytoma therapeutics.

## Simple summary

Malignant astrocytoma patients have poor clinical outcomes, and novel treatments are needed to limit tumor recurrence and improve their overall survival. These tumors have a malignant phenotype mediated by altered mitochondrial metabolism, abnormal protein processing, and adaptation to hypoxia. We have previously published that astrocytomas are especially vulnerable to proteasome inhibitors as well as to inhibitors of the mitochondrial Lon Peptidase 1 (LonP1), but the effect of combining the two strategies has not been reported. Here, we present evidence that the dual inhibition of LonP1 and Chymotrypsin-like (CT-L) proteasome activity effectively induces cellular reactive oxygen species (ROS) production, leading to apoptosis in malignant astrocytoma established cell lines and patient-derived glioma stem cell-like cultures. We developed BT317, a small molecule dual inhibitor, which crosses the blood-brain barrier and shows strong synergy with the standard of care, temozolomide (TMZ), in the astrocytoma cell lines independent of their isocitrate dehydrogenase (IDH) profile and in an orthotopic glioma murine model. This preclinical study demonstrated the potential of dual LonP1 and CT-L proteasome inhibition as a novel therapeutic strategy for malignant astrocytoma and provides insight for future clinical translational studies alone or in combination with other chemotherapies.

## 1. Introduction

Malignant astrocytomas, including glioblastoma (GBM, grade 4 IDH wildtype astrocytoma), are the most aggressive brain tumors, with a less than three-year expected survival rate following surgical resection, radiation, and chemotherapy [1]. The current standard of care, temozolomide (TMZ, temodar), an alkylating agent, provides a median survival advantage of 2.5 months for GBM patients when added to surgery and radiation therapy [2]. Despite significant efforts and numerous phase 3 clinical trials utilizing molecular targeted therapies and immunotherapy approaches, developing safe and efficacious therapies for malignant astrocytomas has been exceedingly difficult. In the past 16 years, only three therapies (TMZ, bevacizumab, TTFields) have been approved for the treatment of astrocytomas [3]. Thus, there is an urgent need to develop novel therapeutic strategies.

Malignant astrocytomas are currently classified based on their genetic and epigenetic profiles like isocitrate dehydrogenase 1 or 2 (IDH1/2) mutant/wildtype, Alpha-Thalassemia X-linked Intellectual Disability (ATRX) ATRX (retention/loss), and p53 (mutant/wildtype) [6]. Less than 10 % of grade 4 astrocytomas are IDH mutant, which are associated with an improved response to treatment and prognosis compared to the IDH wildtype tumors [4]. Based on these biologic and survival differences, the most recent WHO glioma classification excludes tumors with an IDH mutation from being classified as GBM and refers to such tumors as astrocytoma grade 4, IDH mutant [5]. Even though the presence of the IDH mutations predicts a better outcome, the survival of patients with grade 4 IDH mutant astrocytoma treated with the standard therapy (radiation and TMZ) is still only 31 months [6] as compared with the GBM patients with a IDH wildtype status which survive an average less than 24 months [7]. Due to differences in genetic profile, response to treatment, and overall survival, it is imperative to include different types of malignant astrocytoma models with varying genetic mutations in the efforts to develop novel therapies.

The invasive phenotype of malignant astrocytomas is partly mediated by overexpression of the transcriptional activator hypoxia-inducible factor 1 alpha (HIF-1α). HIF-1α contributes to the hypoxia-driven maintenance of glioma stem-like cells (GSCs) [8] by supporting self-renewal [9], angiogenesis [10], increased invasiveness [11], and high levels of genetic instability. The latter contributes to tumor heterogeneity and presents a significant challenge in devising novel therapeutic strategies for its treatment [12]. There are hundreds of gene targets in the HIF-1α signaling pathway, but HIF-1α directly upregulates only a small number of genes, including the nuclear-encoded Lon Peptidase 1 (LonP1) [13]. LonP1 is an ATP-dependent protease that regulates mitochondrial homeostasis through its three main functions: (1) proteolytic degradation of mitochondrial proteins (including Aconitase and Transcription Factor A Mitochondrial, TFAM) [13], (2) protein chaperone [14,15], and (3) mitochondrial DNA (mtDNA)-binding protein [16].

LonP1 overexpression is known to promote invasion and metastasis and correlates with poor outcomes in multiple cancers, including colorectal [17], melanoma [17], and oral cancers [18]. Our previous results showed that LonP1 is overexpressed in astrocytomas, and its elevated expression levels are associated with high tumor grade and poor survival [19]. Furthermore, LonP1 knockdown in established human glioma lines, D-54 MG and U-251 MG, reduced cell viability under normal conditions and drastically impaired survival under hypoxic conditions [19]. This is concomitant with a decrease in mitochondrial respiration. Notably, LonP1 pharmacological inhibition using the coumarinic compound 4 (CC4) [20] inhibited glioma cell proliferation and synergistically enhanced the therapeutic efficacy of TMZ *in vitro* [19].

There is difficulty in generating specific LonP1 inhibitors [20], which is attributable to its structural similarities with other known proteases and especially the proteasome [21]. However, targeting the chymotrypsin-like (CT-L) proteasome activity may be beneficial, as it plays an essential role in tumor cell survival [22] and treatment resistance [23]. Bortezomib (BTZ) exhibits dual LonP1 and CT-L inhibition and possesses a boronic acid group that can transform into a boronate ester when exposed to reactive oxygen species (ROS) [24]; however, its major limitation is poor blood-brain barrier (BBB) permeability [25]. In the subcutaneous U-87 MG and U-251 MG glioma models, BTZ was found to sensitize the glioma tumors to TMZ by suppressing Forkhead Box M1 (FOXM1) mediated treatment resistance. The FOXM1 was markedly up-regulated in established TMZ-resistant glioma cell lines and HGG patients, related to poor prognosis [26]. Recent work in multiple myeloma has demonstrated the strong synergy with LonP1 and CT-L proteasome inhibition [27], using two specific inhibitors previously shown to have no cross activity and to target LonP1 protease, Bardoxolone methyl (CDDO-ME) [28,29], and to target CT-L proteasome activity, carfilzomib (CFZ) [30] respectively, were employed. Thus, bortezomib, which inhibits the proteasome, can also inhibit LONP1, albeit at higher concentrations. By contrast, CFZ inhibits the proteasome but not LONP1 or CLpXP, which is another mitochondrial ATP-dependent protease. Essentially, it is worth mentioning that CDDO derivatives are not LonP1-specific and have multiple cellular targets. CDDO derivates have been known to have binding sites for Jak1 and Stat3 [31], IκB kinase beta (IKK-β) [32] and Keap1 [33]. However, CDDO derivatives offer great prospects for recognizing the specific allosteric binding site(s) within this key AAA^+^ protease and strengthens the discovery of specific, high affinity compounds which can successfully inhibit LonP1.

We now report that there is a strong synergy between LonP1 and CT-L proteasome inhibition in established and patient-derived malignant astrocytoma cell lines. Furthermore, we identified BT317, a derivative of Coumarinic Compound 4 (CC4) [19,20], and a dual LonP1 protease and CT-L proteasome inhibitor as an agent with selective anti-glioma activity. Combinations of genetic and epigenetic profiles where loss or mutation of certain gene(s) such as p53, IDH1/2 and or ATRX serves as an essential hallmark of certain gliomas subtypes. Alpha-Thalassemia X-linked Intellectual Disability (ATRX) gene plays an important role in maintaining telomere length and genomic integrity and its mutation or loss is a hallmark of glioblastomas and astrocytomas. Here, we have selected established and patient-derived astrocytoma lines with various combinations of genetic and epigenetic profiles such as isocitrate dehydrogenase 1 or 2 (IDH1/2) mutant/wildtype, ATRX (retention/loss), and p53 (mutant/wildtype), to examine BT317 target specificity against multiple astrocytoma subtypes. We then investigated the efficacy of BT317 as a single agent and in combination with TMZ as a therapeutic strategy for malignant astrocytoma, including a glioma orthotopic xenograft model.

## 2. Methods

### Ethics Statement:

All astrocytoma specimens were collected under an institutional review board review (IRB) approved protocol from patients who underwent surgical tumor resection at the University of California, Irvine Medical Center. A specialized neuropathologist completed the neuropathological review, using the 2021 WHO Classification of Tumors of the Central Nervous System [34]. All animal studies were performed following the guidelines established by the Institutional Animal Care and Use Committee (IACUC) at the University of California, Irvine.

### Synthesis of Small molecule BT317 and Related Compounds:

Synthesis of BT317 was initiated using compound D (methyl 6-chloro-2-oxo-2H-chromene-3-carboxylate) (Fig. 2C). A detailed stepwise protocol is listed in the supplementary section under “Synthesis of Small molecule BT317 and Related Compounds”.

### Primary Glioma/Astrocytoma Stem Cell-like Cultures (GSC):

Patient-derived GSC were isolated from surgical astrocytoma samples in the laboratory of Dr. Daniela A. Bota using a previously published method [35]. The surgical specimens were procured in line with WHO Guiding Principles on Human Cell, Tissue and Organ Transplantation. The GSC cultures (DB81 and DB93) were maintained as non-adherent neurospheres in Neurobasal medium (Thermo Fisher) supplemented with 20 μg/mL EGF (Thermo Fisher), 20 μg/mL FGF (Thermo Fisher), B27 (Life Technologies), GlutaMAX (Thermo Fisher), 5 mM sodium pyruvate (Thermo Fisher), and 1 % penicillin/streptomycin (Thermo Fisher). Each patient-derived cell line was expanded, cryopreserved as low-passage stocks, and tested for mycoplasma using a MycoScope PCR Mycoplasma Detection Kit (Genlantis). All the patient-derived cell lines were used for the initial ten passages.

### Established Human Glioma Cell Lines:

Five established adult astrocytoma cell lines (U-251 MG, D-54 MG, U-87 MG, T98G, and HOG) and the pediatric astrocytoma CHLA-200 cell line were maintained in DMEM/F-12 medium (Corning) containing 292 μg/mL glutamine, 1 % penicillin/streptomycin (Thermo Fisher), and 10 % FBS (Invitrogen). For specific experiments (such as establishing the HOG intracranial xenograft model), the HOG cells were propagated in GSC-like media, as indicated above. All the cell cultures were maintained at 37°C and 5 % CO_2_ in a humidified incubator.

### Normal Tissue Cell Lines:

Human astrocytes were purchased from ScienCell Research Laboratories and were maintained in astrocyte culture medium containing astrocyte growth supplement (AGS, Cat #1852), 2 % fetal bovine serum, and 1 % penicillin/streptomycin. The human mammary gland epithelial adherent non-tumorigenic cell line MCF-10A was maintained in DMEM/F-12 medium containing 0.5 mg/mL hydrocortisone, 20 ng/mL EGF, 100 ng/mL cholera toxin, 1 % penicillin/streptomycin, and 5 % horse serum (Life Technologies). The human lung fibroblast line HPF242 was maintained in DMEM/F-12 containing 10 % FBS and 1 % penicillin/streptomycin. All cell cultures were maintained at 37°C and 5 % CO_2_ in a humidified incubator. Unlisted reagents were purchased from Sigma-Aldrich and Thermo Fisher Scientific.

### Generation of Transgenic Astrocytoma Cell Lines:

For ectopic expression and genetic knockdown, cells were transfected with a vector control or full-length LonP1 using a lentiviral system. shRNAs were obtained from Horizon: nontargeting (shCTRL, VSC11651) and LonP1 knockdown (sh-LonP1.1: 10776083, sh-LonP1.2: 5672171, and sh-LonP1.3: 226914271). These LonP1 knockdowns are doxycycline inducible. The LonP1 overexpressing (LonP1: OHS5898-202622213), and vector control (OHS5832) clones were ordered from Horizon. HOG cells were transfected with the lentivirus plasmids and sorted to 100 % purity by FACS following ZsGreen1 expression with a BD FACSAria II cell sorter. Cells were subjected to Puromycin selection and selected for the positive population. The knockdown was verified by Western blot.

#### Cell Viability Assays:

Astrocytoma cells were seeded at a density of 10,000 cells per well in a 96-well plate (n = 4 replicates per condition). The following day, equal volume of the drugs dissolved in DMSO were added to each well at the specified concentrations indicated in the figure legends. After 5 days, 25 μL of XTT Cell Viability Assay solution (Biotium) was added. After 4 h, the absorbance was measured at 490 nm using a SpectraMax Plus 384 microplate reader. GraphPad was then used to perform a log transformation and generate a nonlinear regression curve to calculate IC_50_ viability. The Biochemically Intuitive Generalized Loewe Model (BIGL) was used to determine agonism or antagonism (https://cran.r-project.org/web/packages/BIGL) [36]. BIGL as a tractable and flexible approach to comprehensively analyze drug interactions while evaluating combination therapies with minimal and consistently available data requirements, that naturally generalizes the widely used Loewe model and retains Loewe’s biochemical interpretability.

### Apoptosis Analysis by Flow Cytometry:

Established and patient-derived astrocytoma cells (10^5^ per condition) were treated with CDDO-ME and CFZ alone or in combination, and BT317 and TMZ alone or in combination, as stated in the figure. The cell apoptosis was analyzed by flow cytometry after AnnexinV-FITC/PI staining using apoptosis detection kit (ProteinTech). At the end of treatment incubation, all the cells were collected, washed with 1X PBS twice and resuspend in 100 μL of 1 × binding buffer supplied in the kit. Add 5 μL of CL488-Annexin V and incubate for 15 minutes at room temperature in the dark. After the incubation period, added 400 μL of 1x PBS ito each tube and centrifuged at 300 × g’s for 5 min at 4 °C to collect cells. Resuspend cells back in 500 μL of 1x PBS with propidium iodide (PI) staining solution containing 50 μg/mL PI and 100 μg/mL DNase-free RNaseA and incubated for 5 minutes at room temperature. After the incubation period, centrifuged at 300 × g’s for 5 min at 4 °C to collect cells. Cells were resuspended in 500 μL of 1x PBS and were subjected to fluorescence-activated cell analysis of DNA content. The percentage of cells with subdiploid DNA content was taken as a measure of the apoptotic rate of the cell population. Flow cytometry was performed on a BD Fortessa X-20 (BD) analyzer, and the data were analyzed using FlowJo (Treestar) software.

### Reactive Oxygen Species Quantification Assay:

Established and patient-derived astrocytoma cell lines were plated and treated for the indicated time period. At the end of incubation, the CellROX^™^ Deep red Reagent (Thermo Fisher) was added at a working concentration of 5 μM for 30 minutes to the culture media. After three 1X PBS washes, the cell samples were stained for DAPI for nuclear staining for 5 min, and flow cytometry was performed on a BD Fortessa X-20 (BD) analyzer. The data were analyzed using FlowJo (Treestar) software. ROS levels are quantified as the relative mean intensitity.

### Sphere Formation Assay:

Genetically modified HOG cells, with LonP1 over-expression and LonP1 downregulation, and the scrambled controls were exposed to doxycycline for 96 h; then the cells were washed with HBSS and incubated with Accutase (Sigma) for 3 min at 37 °C to create a single-cell suspension. 1 mL of the methylcellulose stock media was added (R&D Systems) to a 24-well non-treated plate followed by 1 mL of culture media containing 4000 cells. The cells were gently mixed by swirling with 200 μL pipette tips until they were uniformly distributed without introducing bubbles in the well. After 14 days of incubation, images of tumor spheres were captured and quantified using ImageJ (NIH).

### Western Blotting:

Astrocytoma cells were exposed to the indicated drugs at the specified time, followed by lysis with RIPA lysis buffer containing 1 mM PMSF, 1 mM Na_3_VO_4_, and a protease inhibitor cocktail (Sigma). The protein concentration was standardized using the DC Protein Assay (Bio-Rad) with a SpectraMax Plus 384 microplate reader. A Precision Plus Protein Kaleidoscope^™^ ladder (Bio-Rad) and approximately 20 μg of sample were loaded onto each well and run on a Mini Protean TGX Gel (Bio-Rad) before being transferred to an Immobilon Transfer Membrane (Millipore). The primary and secondary antibodies used were 1:2000 LonP1 (Proteintech, 15440-1-AP), 1:1000 Aconitase2 (Abcam, ab71440), 1:4000 GAPDH (*GeneTex;* GTX100118), 1:1000 TFAM (Fisher Scientific, PA5-80107), 1:4000 β-Actin (Novus Biologicals; NB600-501), 1:10,000 goat anti-mouse IgG F(ab’)2 (Enzo Life Sciences, ADI-SAB-100-J), and 1:3000 IgG (H+L) Goat anti-Rabbit HRP (Invitrogen, 32460); these were used according to the manufacturer’s recommendations and diluted in TBST with 5 % BSA. Chemiluminescence was visualized using Amersham^™^ ECL^™^ Prime western blotting Detection Reagent (GE Healthcare) and imaged using an Azure c600 Molecular Imager. ImageJ was used to align the bands, improve contrast (<20 %), and normalize and quantify all bands.

### Mitochondria Isolation:

1 × 10^9^ HOG cells were suspended in a homogenizing buffer (225 mM mannitol, 75 mM sucrose, 0.5 mM EDTA and 25 mM HEPES, pH 7.4). The cells were passed through a 25-gauge needle with a 1 mL syringe for homogenization at a consistent speed of 10 stocks with 2 mins breaks for a total of 4–6 times, throughout on ice. Multiple rounds of centrifugation at 2000 rpm for 5 mins were performed to pellet the unbroken cells and nuclei, plasma membranes, lysosomes, microsomes, and cytosol, respectively. The supernatant from these steps was centrifuged at 8000 rpm for 10 mins to pellet crude mitochondria. This step was repeated twice in the homogenizing buffer to remove any microsomal contamination. The crude mitochondrial pellet was suspended in 1 % digitonin lysis buffer (1 % digitonin, 20 mM Tris-HCL, 50 mM NaCl, and 0.1 mM EDTA). After a 30 min incubation on ice, the lysates were centrifuged at 12,000 rpm for 10 min, and the mitochondrial fraction was collected frozen at −80 °C until use for further LonP1 protein purification using immunoprecipitation protocol.

### LonP1 Purification Using Immunoprecipitation:

The Pierce Crosslink IP KThermo Scientific, 26147) was used to capture the LonP1 antibody (Proteintech, 15440–1-AP) to Protein A/G Agarose magnetic resin and to covalently immobilize it to the support by crosslinking it with disuccinmidyl suberate (DSS). Further, the LonP1 antibody resin mix was incubated with the mitochondrial fraction lysate from the above protocol that contains the protein antigen of interest (LonP1), allowing the antibody: antigen complex to form. The antibody: antigen complex were collected after washing to remove nonbound components of the sample. LonP1 protein was recovered by dissociation from its antibody complex with the elution buffer supplied in the kit. The entire procedure was performed using a magnetic Eppendoff stand, allowing solutions to be fully separated from the magnetic agarose resin. LonP1 elution specificity and purity was confirmed by Tris-glycine SDS gel and Western blot.

### LonP1 Protease Activity Assay:

LonP1 protease activity was analyzed using a Pierce Fluorescent Protease Assay Kit (Thermo Scientific^™^, 23266). The mitochondrial isolation step was introduced to enrich the LonP1 fractions. The LonP1 immunoprecipitation was performed with the A/G magnetic bead crosslinked LonP1 antibody from the total mitochondrial lysate. Purified 200 nM LonP1 or equivalent total mitochondrial lysate and 10 mM MgCl_2_ were prepared in BupH^™^ Tris-buffered saline. LonP1 inhibitors or vehicle (DMSO) were then added, and the samples were incubated at 37°C for 1 h. Following this incubation, 4 mM FITC-casein (Thermo Scientific^™^) with or without 2 μM ATP (Thermo Fisher) and 10 mM MgCl_2_ were added prior to measurement using a Biotek Synergy HT plate reader. Digestion of fluorescein-labeled casein was assessed by measuring fluorescence with excitation and emission filters at 485 and 534 nm, respectively.

### Proteasome Activity Assay:

The proteolytic activity was assessed in cell, whole blood, and tissue lysates using the Proteasome-Glo^™^ 3-Substrate System (Promega, G8531), comprising Proteasome-Glo^™^ Chymotrypsin-Like Assay, Proteasome-Glo^™^ Trypsin-Like Assay, and Proteasome-Glo^™^ Caspase-Like Assay. D-54 MG cells were seeded at a density of 200,000 cells per well onto a 6-well plate and subsequently exposed to the indicated inhibitors (BT317, BT395, BT397, BT399) at the specified concentrations and endpoints. Proteasome isolation from blood samples was done within 48 h after blood collection. Heparinized whole blood was centrifuged at 2000 rpm for 10 min at 4°C to remove plasma. The pelleted blood cells were washed with ice-cold PBS at 2000 rpm for 10 min at 4°C. The pelleted whole blood cell samples were stored at −80°C until further processing for proteasome activity evaluation. Brain tissue samples were flash-frozen and then grinded into fine powder using a cryogenic tissue grinder with dry ice (Fisher), and stored at −80°C. All samples were lysed in cold lysis buffer (50 mM HEPES at pH 7.4, 250 mM Sucrose, 5 mM MgCl_2_, 0.5 mM DTT, 40 mM KCl), mixed, and incubated on ice for 30 min with vertexing at 10-minute intervals. The samples were then centrifuged at 14,000 rpm for 10 min at 4°C. The supernatants were collected and mixed 1:1 with a stabilizing buffer (40 mM HEPES at pH 8.0, 1 mM EDTA, and 20 % glycerol) in deionized, sterile H_2_O. The samples were then stored at −80°C for at least one hour. The protein concentration of each sample was standardized using the DC Protein Assay (Bio-Rad) with a SpectraMax Plus 384 microplate reader. Samples were then diluted with ice-cold proteasome dilution buffer (10 mM HEPES at pH 7.6) and plated in a black, clear, flat-bottom 96-well plate at a concentration of 8 μg protein in 50 μL/well (n = 3–4 replicates/sample). Then 50 μL of Proteasome-Glo^™^ reagent was added to each well. The plates were placed on a plate shaker at 300–500 rpm for 30 sec, followed by 10–30 min incubation at room temperature. Luminescence was read using a Biotek Synergy HT plate reader.

### BT317 Maximum Tolerated Dose (MTD) Finding and Brain Distribution in Mice:

100 mM BT317 and 100 mM TMZ were reconstituted in DMSO, and were further diluted in 1X PBS, at a final volume of 500 μL to generate the correct dosage for intraperitoneal injection (i.p.) in 10–14-week-old Rag1 KO immunodeficient mice (Jackson Laboratory, B6.129S7-Rag1^tm1Mom^/J). Mice were monitored following injection, and the clinical score was determined based on activity, appearance, and body condition with a maximum score of 3, which was necessary to define the MTD [37]. A contract research organization (Cyprotex and Biotechnology Innovation and Optimization Center) performed mass spectrometry on flash-frozen brains and tail vein blood samples to measure BT317 concentration in the brain and plasma.

### HOG Intracranial Xenograft Mouse Model:

Mice of both sexes were randomly assigned to receive intracranial transplantation of 20,000 and 50,000 HOG cells into the right frontal lobe of 10–14-week-old Rag1 KO immunodeficient mice (Jackson Laboratory, B6.129S7-Rag1^tm1Mom^/J) in early and late tumor models, respectively. In the early model, 5 days post-tumor intracranial implantation, 100 mg/kg BT317, intraperitoneal (i.p.) was administered daily (for 5 consecutive days) or every other day (for 10 days) for a total of 5 doses, as specified. In late model, 10 days post-tumor implantation, 100 mg/kg BT317 ± 100 mg/kg TMZ i.p. was administered daily (for 5 consecutive days) or every other day (for 10 days) for a total of 5 doses, as specified. Sample sizes of n = 6 was estimated from power analysis based on preliminary measurement of variability and desired statistical power (π=0.80) and significance criterion (p < 0.05) using ANOVA, as well as our past experience with similar analysis. Animals were observed daily and sacrificed upon observation of distress, including hemiparesis, obtundation, hunchback, or a greater than 20 % weight loss from the maximum weight achieved.

### Statistical Analysis:

When appropriate, data were analyzed using either Student’s *t*-test or log-rank (Mantel-Cox) test. Data are presented as mean ± standard error of the mean (SEM), and significance levels were set at 0.05. Significance between groups is denoted by *P < 0.05, * *P < 0.01, * **P < 0.001. Data were analyzed using the GraphPad Prism 5.0 software (GraphPad Software, La Jolla, CA, USA). For the XTT viability assays, raw data were processed using a log transform and a dose-response inhibition nonlinear model to determine the IC_50_ and standard error. The statistical significance of the Kaplan-Meier survival curve was verified using the Mantel-Cox log-rank test.

## 3. Results

### 3.1. Dual targeting using specific LonP1 and CT-L proteasome inhibitors enhances cytotoxicity in established and patient derived astrocytoma cell lines

Proteasome inhibition has emerged as a compelling concept in oncology, with strong clinical selective anti-tumor activity observed in various malignancies [27–29]. Mitochondrial metabolism and response to free radical damage is extensively regulated by mitochondrial protease LonP1, encoded by the nuclear gene LONP1. Evidence from a multiple myeloma study showed that LonP1 is upregulated in response to proteasome inhibition and that increased LonP1 levels confer partial resistance against proteasome inhibition [27]. The synthetic triterpenoid CDDO-Me is a specific inhibitor of LonP1 protease that does not interfere with proteasome acitivity [28,29]. Carfilzomib (CFZ), an FDA approved proteasome inhibitor used for the treatment of multiple myeloma, is highly selective for the chymotryptic activity of the proteasome and has no effect on the LonP1 enzymatic activity [30,38].

**Fig. 1.**
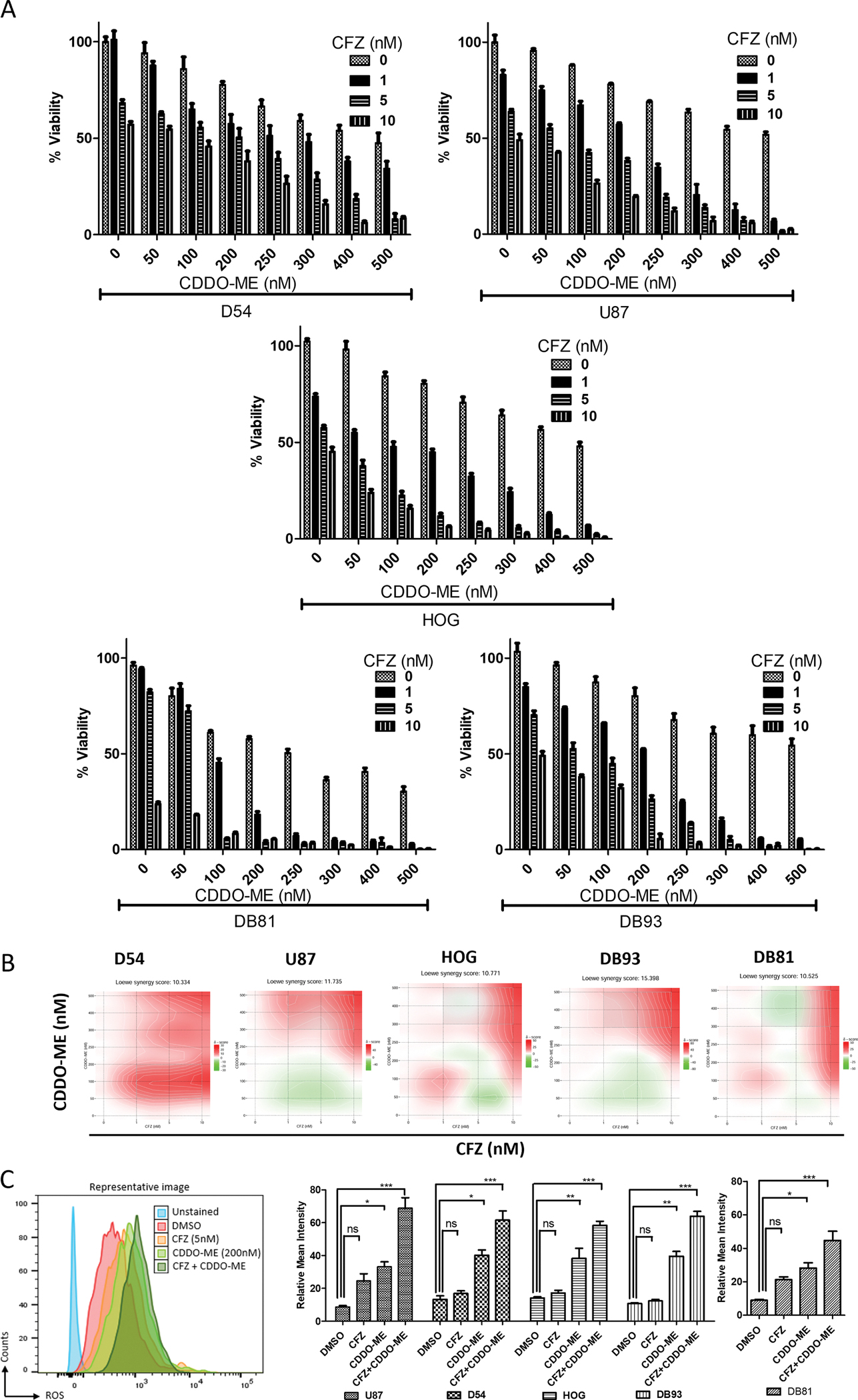
Dual LonP1 and Chymotrypsin-like Proteasome Inhibition Has Synergy in Malignant Astrocytoma and Shows Enhanced ROS Production. A. Established astrocytoma lines D54-MG, U87, HOG, and primary patient-derived astrocytoma lines DB93 and DB81 were incubated with the indicated concentrations of CDDO-ME ± CFZ, for 48 h. Annexin PI+ cells were quantified as a measure of cell viability at the end of the incubation period by flow cytometry. Error bars indicate the standard deviations of three biological replicates. n = 3 technical replicates from 3 biological replicates for each group. B. Synergy test between CDDO-ME and CFZ was evaluated using BIG, with red representing significant synergy and green representing anergy. Established (D54-MG, U87, HOG) and primary (DB93 and DB81) astrocytoma cells were incubated with the indicated concentrations of CDDO-ME ± CFZ (0, 1, 5 and 10 nM), for 48 h. The cell viability data from the flow cytometry was used to interpret synergy. n = 3 technical replicates from 3 biological replicates for each group. A synergy test between CDDO-ME and CFZ combination was performed with the XTT data using online tool SynergyFinder - Documentation (fimm.fi). C. ROS levels were assessed following treatment with 5 nM CFZ and/or 200 nM CDDO-ME at 8 h timepoints in D54-MG, U87, HOG, DB93 and DB81 astrocytoma cell lines. Statistical significance was determined by *t*-test. * P < 0.05, * *P < 0.01, * **P < 0.001; ns, not significant.

Here, we demonstrated that the LonP1 inhibitor, CDDO-ME [28], and CT-L proteasome inhibitor, CFZ [28,29], have strong synergy in reducing viability of multiple established and patient-derived malignant astrocytoma cell lines (Fig. 1A and B). Annexin V CoraLite 488 and PI staining in flow cytometry assay results showed that our patient-derived malignant astrocytoma cell lines are sensitive to CDDO-ME and CFZ. Interestingly, established and primary patient-derived malignant astrocytoma lines were found more sensitive to combination treatment with CDDO-ME and CFZ than either drug alone (Fig. 1A). Drug combination dose response analyses of CDDO-ME and CFZ using the Biochemically Intuitive Generalized Loewe Model (BIGL), showed that these two drugs work in synergy, where a low dose of each drug in combination is more effective than individual effector drug doses (Fig. 1B). We also performed an XTT viability assay to determine the IC_50_ of CDDO-ME and CFZ alone and in combination in various established and primary malignant astrocytoma cell lines (Supplementary Fig. S1A–B). These flow cytometric results were further confirmed and validated through the XTT assay. We found that IC_50_ for CDDO-ME ranges between 169 and 320 nM among various malignant astrocytoma cell lines, and the cell viability is significantly decreased by ~ 6–20 fold when CDDO-ME is used in combination with 5 nM CFZ (Supplementary Fig. S1A–B). The 200 nM CDDO-ME and 5 nM CFZ concentrations used in all the subsequent combination studies were chosen after initially determining the dose range for synergy in combination (Fig. 1A). All malignant astrocytoma lines used in this study showed similar sensitivity to the CDDO-ME and CFZ combination in the two independent assays. Since accumulation of reactive oxygen species (ROS) is an early indicator of cellular apoptosis, we also performed a flow cytometric analysis to evaluate the timeline of ROS increase upon CDDO-ME treatment. We found that ROS production gradually peaks at 8 h after CDDO-ME exposure in all four cell lines, after which it decreases in 12 h and 24 h in D54 and U87 cells while DB93 cells shows stable levels of ROS even at 24 and 48 h after CDDO-ME treatment (Supplementary Fig. S1C). Therefore, we chose the 8 h time point for all the subsequent studies to evaluate ROS induction following CDDO-ME ± CFZ exposure. A significant increase in ROS (by ~50 %) was observed following exposure to 200 nM CDDO-ME + 5 nM CFZ (Fig. 1C) as opposed to either CFZ or CDDO-ME alone in all established and patient-derived malignant astrocytoma cell lines. These findings suggest that LonP1 and CT-L inhibitors work synergistically in reducing cell viability by inducing ROS and apoptotic cell death in our *in vitro* system. Therefore, targeting both the proteasome and mitochondrial protease LonP1 may be beneficial for treatment of malignant astrocytoma.

### 3.2. Structure activity relationship modeling for developing CC4 derivatives

**Fig. 2.**
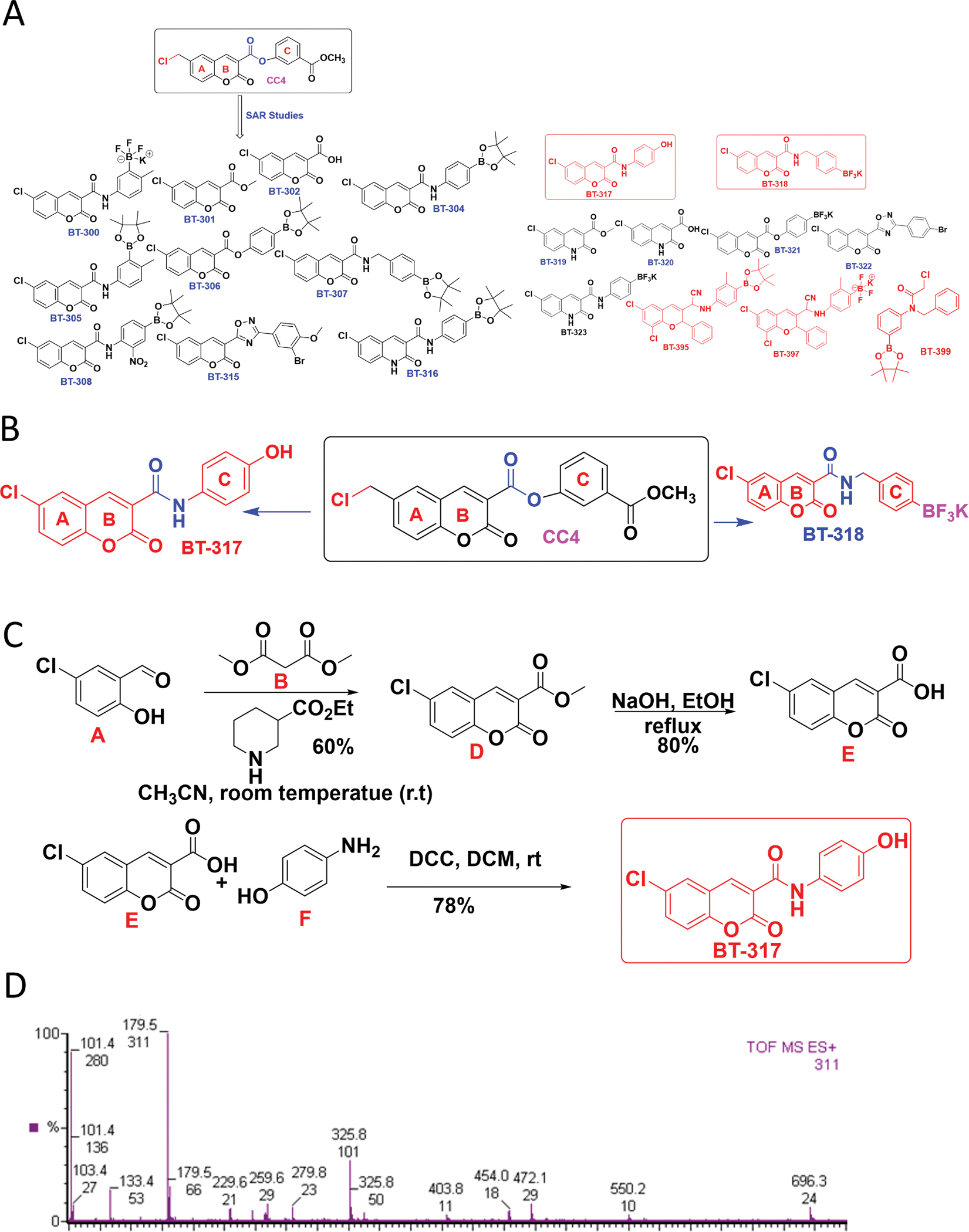
LonP1 Small Molecule Inhibitor BT317 was Derived from CC4. A. SAR studies of compound CC4 (total 19 compounds). B. Target-to-hit compounds BT317 and BT318. C. Synthesis of BT317 and D. demonstrable synthesis shown at 325.8 molecular weight using liquid chromatography mass spectrometry.

Our previous work with Coumarinic Compound 4 (CC4) demonstrated that LonP1 pharmacological inhibition inhibits astrocytoma cell growth, adaptation to hypoxia, and increases the anti-tumor efficacy of TMZ [19]. However, previously published coumarin compounds have been found to exhibit high hepatotoxicity, which limits their potential translation into humans [39]. To decrease off-target toxicity and improve anti-tumor efficacy, we derived four novel LonP1 inhibitors from CC4 using structure-activity relationship (SAR) modeling. First, we synthesized 19 compounds using SAR (structure activity Relationship) studies. The parent compound CC4, contains an ester group, which is very prone to hydrolysis *in vivo*, so to prevent hydrolysis and increase the cell permeability and stability, we synthesized amide derivatives. At ring **A,** CC4 contains benzyl chloride (Fig. 2A), which is very prone to oxidation, so to protect that, we added a chloro group directly to ring **A** in the new compounds to reduce oxidation and increase cell permeability. In compounds BT315 and BT332 we replaced the amide groups with an oxadiazole group to increase stability and solubility. In BT395 and BT397, we replaced the amide group with an amino-nitrile group which is more stable and increases stability. After synthesizing 19 compounds, we tested their biological activities as LONP1 inhibitors though XTT assay and Annexin V and PI assays in flow cytometry. From our biological studies like protease and proteasome activity assays, we found that 4 of those 19 small molecules (BT395, BT397, BT399, and BT317) are active compounds. We used ADMET 3.0 (https://admetlab3.scbdd.com/) to measure the Kd values. All these compounds were identified using a fragmented based drug discovery approach and were identified *in silico,* using customized computational protein structure modeling programs M4T, MMM, Mutate, and SAR with Autodock4, Surflex-Dock, ICM, PESD, and SFC (B. Das, unpublished data) [24,40]. Upon further biochemical studies such as in protease and proteasome assays, we found that BT317 was more potent, as compared to the other compounds, therefore it was selected for further *in vitro* and *in vivo* validation here.

### 3.3. The novel CC4 derivative, BT317, exhibits on-target dual inhibition of LonP1 protease and proteasome enzymatic activity

**Fig. 3.**
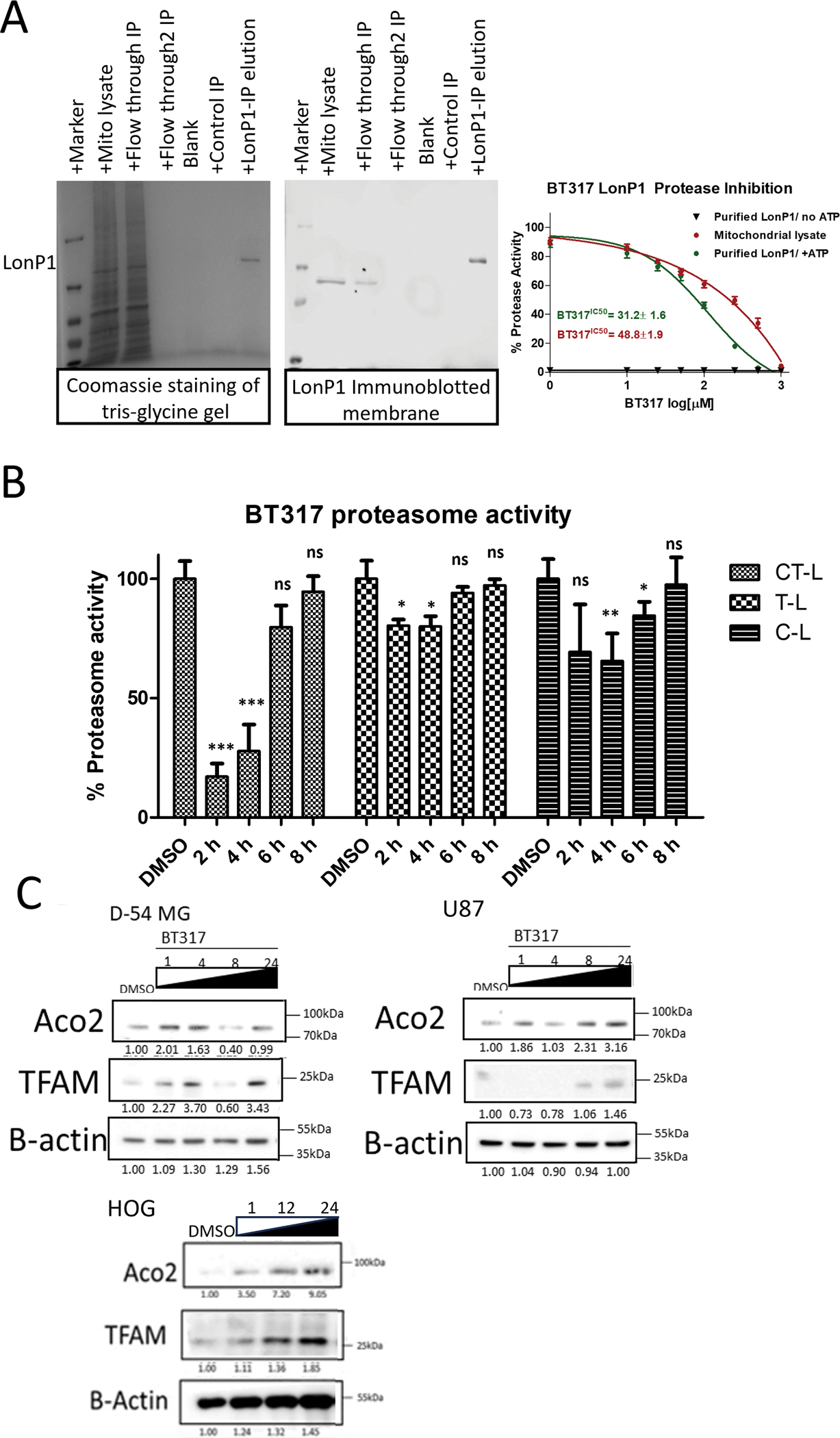
BT317 Acts as a Dual Inhibitor of LonP1 and CT-L Proteasome Activity. A. LonP1 Protease was purified through antibody crosslinking and immunoprecipitated from the mitochondrial lysate of HOG cells, verified on a tris-glycine gel using Coomassie staining (left) and immunoblotted with LonP1 antibody (middle). A gradient concentration of BT317 was added to purified LonP1 protease lysate or equivalent total mitochondrial lysate and incubated for 1 h at 37°C. At the end of incubation, a fluorescent FITC-Casein substrate was added to measure the effect of BT317 on LonP1 enzymatic activity (right). BT317 was assessed for inhibition of protease activity with an IC_50_ = 31.2 μM in purified LonP1 protease whereas total mitochondrial lysate showed the inhibition of protease activity with IC_50_ = 48.8 μM. Omitting ATP from the reaction prevents the overall enzyme activity. The calculations are based on results from three independent experiments. B. 20 μM BT317 was evaluated for proteasome inhibition at 1, 4, 6, 8 h. CT-L= Chymotrypsin-like activity; T-L = Trypsin-like activity; C-L = Caspase-like activity. Statistical significance was determined by *t*-test. * P < 0.05, * *P < 0.01, * **P < 0.001; n.s., not significant. C. The established glioma lines D54-MG, U-87 and HOG were treated with 20 μM BT317 and demonstrated increased Aco2 and TFAM levels. Data is presented as mean ± SEM of at least 3 replicates. Statistical significance was determined by *t*-test. * P < 0.05, * *P < 0.01, * **P < 0.001; n.s., not significant.

Using our structure-activity relationship modeling approach, we derived BT317, BT395, BT397 and BT399 from CC4 and evaluated their relative LonP1 protease and proteasome inhibition profiles. To evaluate the on-target inhibitory potency of the new compounds, we assessed purified LonP1 protease activity inhibition using a FITC-casein substrate conversion in an enzymatic assay (Fig. 3A Supplementary Fig. S2B). We purified the active LonP1 protein from HOG cells overexpressing LonP1 and validated it by SDS-PAGE gel and immunoblotting for LonP1 protein (Fig. 3A). BT317 inhibited purified LonP1 protease activity with an IC_50_= 31.2 μM, while BT397 exhibited similar inhibition at an IC_50_= 34.5 μM. The protease inhibitory activity in total mitochondrial lysates showed similar values, with an IC_50_= 48.8 μM and IC_50_= 51.3 μM with BT317 and BT397, respectively. The protease inhibitory activity of purified LonP1 with BT395 and BT399 compounds showed an IC_50_ values of 50 and 123.8 μM, respectively (Fig. S2A). We further found that removing ATP from the enzymatic reaction resulted in loss of the ATPase dependent LonP1 protease activity, confirming our previously published finding that LonP1 enzymatic activity is ATP dependent [38]. Since many LonP1 inhibitors exhibit dual inhibition of LonP1 and proteasome [20], we next examined whether any of our CC4 analogs can also inhibit the 20S proteasome. For this assay, we prepared extracts from the established malignant glioma cell line, D-54 MG (Fig. 3B). At 10 μM, BT317 yielded a 96 % reduction in CT-L activity, a 27 % reduction in trypsin-like (T-L) activity, and a ~30 % reduction in caspase-like (C-L) activity at 4 h. The proteasomal activity of all three complexes returned to baseline at 8 h post-treatment. The BT395, BT397 and BT399 compounds moderately showed ~40, ~30 and ~50 % inhibition of CT-L activity, whereas BT317 showed ~50, ~30 and ~50 % reduction in T-L and ~50, ~30, ~40 % reduction in C-L at 4 h incubation, respectively (Supplementary Fig. S2B). These results show that, out of the four compounds tested, BT317 is a potent inhibitor of LonP1 protease and CT-L proteasome activity.

BT317 demonstrated quicker kinetic inhibition of CT-L proteasome activity compared to BT395, BT397, and BT399 (Fig. 3B). BT317 showed ~90 % CT-L inhibition at 4 h, suggesting it is a potent inhibitor of CT-L proteasome activity. Given the potent on-target LonP1 protease inhibition of BT317 and its CT-L proteasome inhibition specificity, we selected BT317 for further evaluation as a dual LonP1/proteasome targeted therapy in our astrocytoma models.

The mitochondrial matrix protein aconitase (Aco2) and mitochondrial transcription factor A (TFAM) are known substrates of LonP1 proteolytic activity [31,38]. We examined the effect of BT317-induced LonP1 inhibition on the Aco2 and TFAM protein levels. We treated D-54 MG, U-87 and HOG cells with 20 μM BT317 (Fig. 3C). The D-54 MG line responded with an Aco2 protein level increase of 2-fold at 1 h; which returned to the baseline levels by 24 h. TFAM levels remained elevated by 3-fold at 24 h. In the U-87 line, BT317 resulted in the gradual accumulation of Aco2 to 3-fold and TFAM accumulated by 1.5-fold after 24 h. HOG cells show the 7- and 9-fold accumulation of both Aco2 and TFAM at 12 and 24 h. (Fig. 3C).

### 3.4. BT317 synergizes temozolomide (TMZ) through increased reactive oxygen species production and enhanced apoptosis in patient-derived and established malignant astrocytoma cell lines

**Fig. 4.**
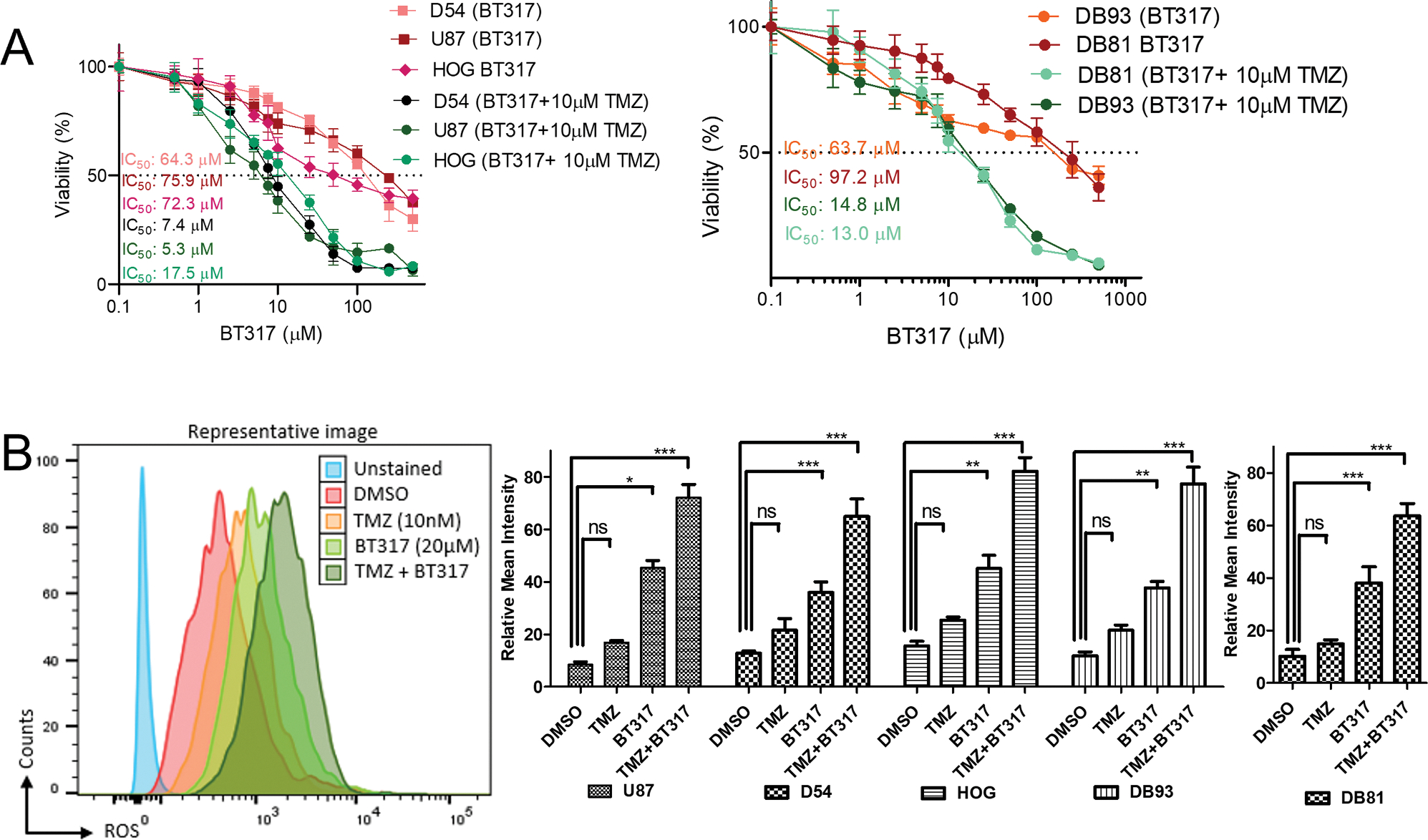
BT317 Drives Cell Death by ROS Production in Established and Patient Derived Malignant Astrocytomas Cell Lines as a Single Agent and Shows Synergistic Activity with TMZ. A. Established (D54-MG, U87, HOG) and primary (DB93 and DB81) astrocytoma cell lines were incubated with the indicated concentrations of BT317 ± 10 μM TMZ, for 120 h. Cell viability was measured at the end of the incubation period. Error bars indicate the standard deviations of three biological replicates. n = 3 technical replicates from 3 biological replicates for each group. B. Patient derived and established astrocytoma cells were incubated with 10 μM BT317 ± 10 μM TMZ, for 8 h. ROS was detected at the end of incubation using deep red oxidative stress reagent through flow cytometry. Relative mean intensities were calculated for each group. Error bars indicate the standard deviations of three biological replicates. n = 3 technical replicates from 3 biological replicates for each group. Statistical significance was determined by 2-way ANOVA. * P < 0.05, * *P < 0.01, * **P < 0.001; n.s., not significant.

Genetic variability is a well-known factor predicting response to therapies both in astrocytoma preclinical models and in human patients. Hence, we selected patient derived and established astrocytoma human cell lines in this study with diverse genetic and epigenetic profiles like isocitrate IDH1/2 (mutant/wildtype), ATRX (retention/loss), and p53 (mutant/wildtype) Supplemental Table 1. Current glioma research also emphasizes the role of GSCs in tumor maintenance, resistance to therapies, and local invasion [41]. The patient derived astrocytoma lines (DB81 and DB93) used in our study were enriched for tumor-initiating GSCs through passaging in serum-free media containing B27 and growth factors that promote stemness (bFGF, EGF), grown in suspension cultures, and have been shown to recapitulate the expression profile of the original patient sample [42]. We determined the IC_50_ values of established and patient derived astrocytoma lines in response to 120 h exposure to BT317 and TMZ at graded doses and Annexin-PI flow cytometry results indicate that 25–50 μM BT317 induces early apoptosis and TMZ enhances the sensitivity to BT317 when given in combination (Fig. 4A and Supplementary Fig. S4B). 10 μM BT317 and 10 μM TMZ concentration used in all the subsequent combination studies was picked after initially screening for viability using flowcytometry of these two drugs where they work synergistically in combination then either of the drug alone (Supplementary Fig. S4B). BT317 had increased toxicity towards the patient-derived GSC lines (Supplemental Table 1) as compared to the established and differentiated astrocytoma lines, consistent with previous research which demonstrated that GSC are more sensitive to proteasome and LonP1 inhibition as compared with the more differentiated established lines [27,43]. BT317 also showed less activity towards the CHLA-200 pediatric line (IC_50_=85.88 μM).

To evaluate normal tissue toxicity, we used a panel of normal cell lines (HPF242 and MCF10A, and human astrocytes). The normal cell lines exhibited a 4–8-fold higher BT317 IC_50_ as compared to the astrocytoma lines, suggesting a good therapeutic index (Supplementary Fig. S3A). We also screened the normal cell line panel to BT395, BT397 and BT399 and the IC_50_ results indicate that these compounds are toxic to the normal cells (Supplementary Fig. S3B.)

Next, we performed a viability study with TMZ alone to calculate the sensitivity of these established and patient derived astrocytoma lines to TMZ and determined an IC_50_ ranging between 23 and 78 μM (Supplementary Fig. S4A) individually mentioned on the graph and we selected 10 μM TMZ which is lower than the IC_50_ dose used for the BT317 combination treatment in the further experiments. Furthermore, BT317 synergized with 10 μM TMZ to decrease cell viability, making the astrocytoma cell lines 5–6-fold more sensitive to the combination treatment compared to BT317 alone (Fig. 4A). The interactive analysis and visualization dose combination response data of BT317 and TMZ generated through Biochemically Intuitive Generalized Loewe (BIGL), showed that the two drugs work synergistically, where a significantly low dose of each drug in combination is more effective than individual effector drug doses (Supplementary Fig. S4C).

Since early ROS accumulation is the readout of apoptotic induction, we did a time course (0, 1, 4, 8, 12 and 24 h) treatment with 20 μM BT317 and found that 8 h is the time point where we see a peak increase in the ROS production. With increasing time (12 h and 24 h) ROS levels plateaued then decreased in all cell lines (Supplementary Fig. S5). Thus, we selected 8 h as the time point for subsequent ROS studies with BT317 + TMZ combination treatment. Co-incubation of 10 μM BT317 with 10 μM TMZ in the D-54 MG, U87, HOG and DB93 lines resulted in a significant increase in ROS levels by ~70, ~60, ~80 and ~60 %, respectively (Fig. 4B). BT317 alone induced a significant increase in ROS levels by ~45, ~30, ~40 and ~40 % at 8 h in the D54, U87, HOG and DB93 line, respectively; however, no significant change in ROS levels was observed upon TMZ treatment alone in any of the astrocytoma lines. These data highlight the potential use of BT317 in combination with TMZ to target diverse astrocytoma cells, independent of their IDH, ATRX and p53 genetic profile.

### 3.5. LonP1 expression directly correlates with BT317 sensitivity in astrocytoma cell lines

Our laboratory has previously demonstrated that cellular stress such as serum starvation, hypoxia and conventional therapies for glioma such as radiation and chemotherapy (TMZ) induce mitochondrial LonP1 expression [19]. LonP1 knock-down using siRNA decreases cell viability and pharmacological LonP1 inhibition sensitizes glioma cells to chemotherapy [19]. These previously published findings suggest that glioma cells upregulate LonP1 protease as an adaptive response to stress and to acquire treatment resistance and aggressive malignant characteristics. Hence, the ability to inhibit LonP1 activity by either pharmacological or genetic approaches presents interest as a potential therapeutic for malignant glioma. However, the mechanisms of resistance to LonP1 inhibitors are not yet studied.

**Fig. 5.**
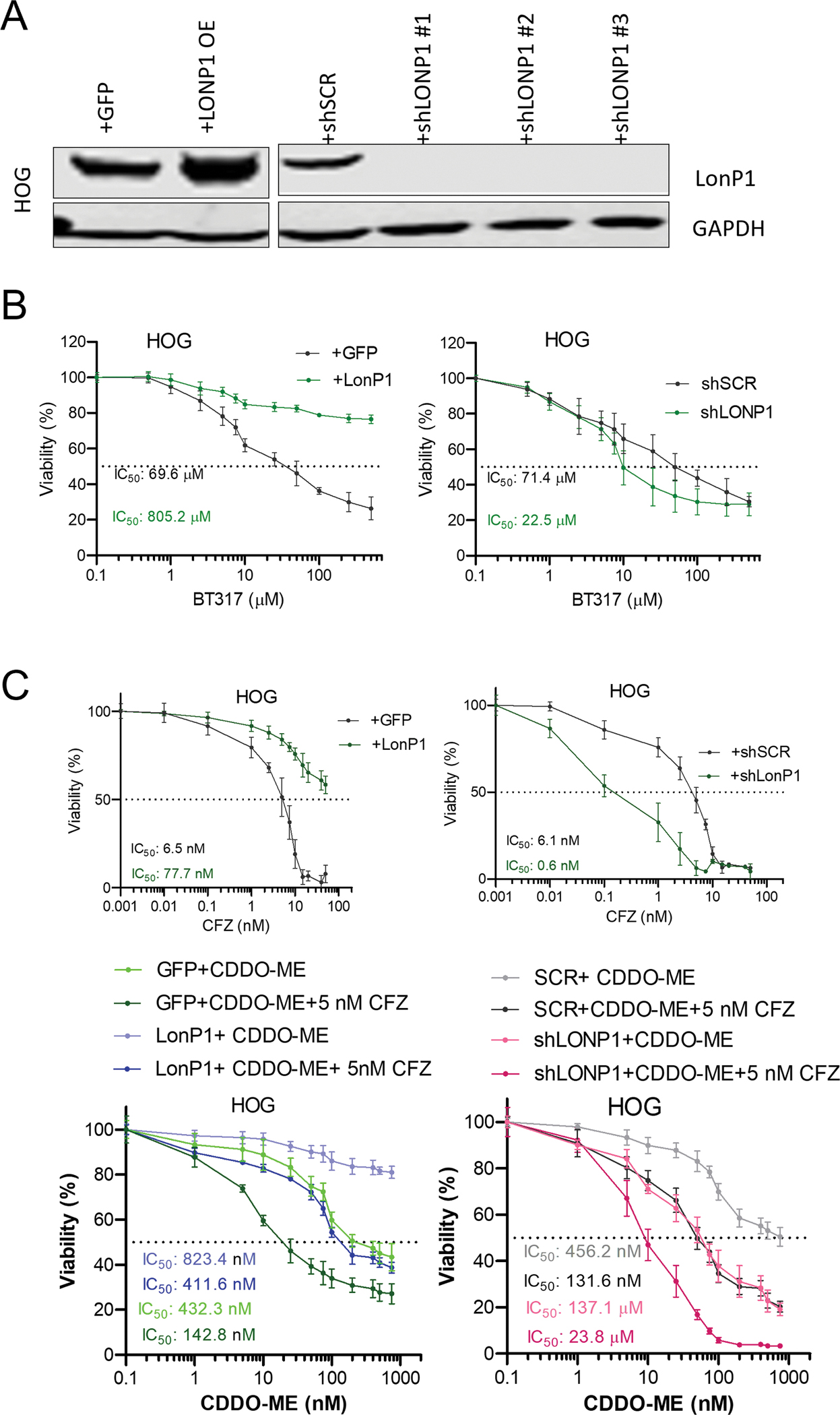

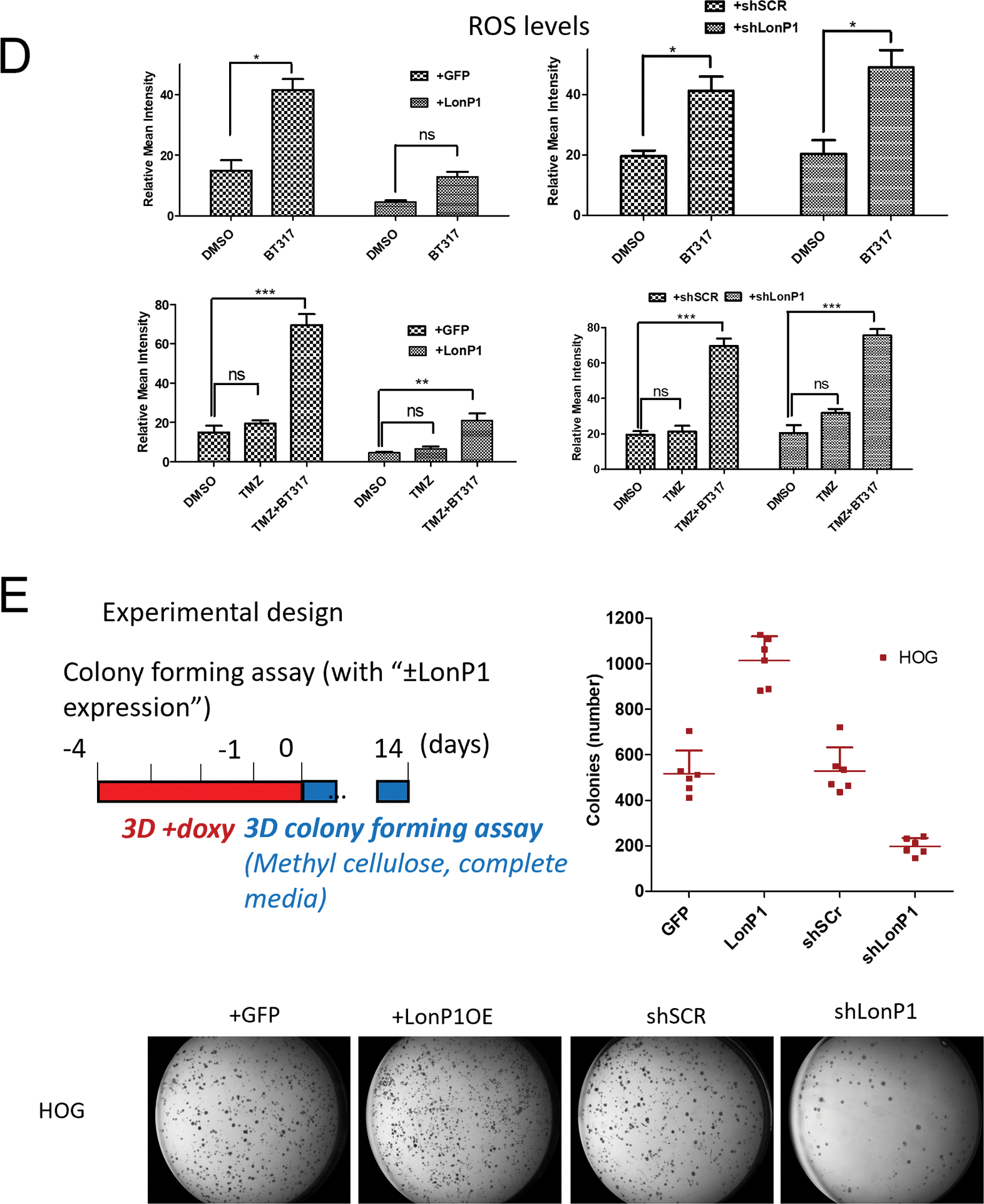
LonP1 is Necessary and Sufficient to Drive Sensitivity to BT317 in LonP1 Overexpression and LonP1 Knockdown Genetic Models. A. Baseline validation of LonP1 protein expressions were shown in cells ± LonP1 overexpression (+LonP1 and +GFP) and cells ± LonP1 knockdown (shLonP1 and shSCR). GAPDH is included as loading control. B. HOG paired lines with LonP1 overexpression (+LonP1 and +GFP) and LonP1 knockdown (shLonP1 and shSCR) were incubated with the indicated concentrations of BT317 for 120 h. Cell viability was measured at the end of the incubation period using XTT. Error bars indicate the standard deviations of three biological replicates. n = 3 technical replicates from 3 biological replicates for each group. C. HOG paired lines with LonP1 overexpression (+LonP1 and +GFP) and LonP1 knockdown (shLonP1 and shSCR) were incubated with the indicated concentrations of CDDO-ME and or Carfilzomib (CFZ) for 120 h. Cell viability was measured at the end of the incubation period. Error bars indicate the standard deviations of three biological replicates. n = 3 technical replicates from 3 biological replicates for each group. D. HOG cells with LonP1 overexpression (+LonP1 and +GFP) and LonP1 knockdown (shLonP1 and shSCR) were incubated with 20 μM BT317 and 10 μM TMZ alone and in combination, for 8 h. ROS was detected at the end of incubation using deep red oxidative stress reagent through flow cytometry. Relative mean intensities were calculated for each group. Error bars indicate the standard deviations of three biological replicates. n = 3 technical replicates from 3 biological replicates for each group. Statistical significance was determined by 2-way ANOVA. * P < 0.05, * *P < 0.01, * **P < 0.001; n.s., not significant. E. Spheroid formation assay: HOG cell line with LonP1 overexpression (+LonP1), LonP1 knockdown (+shLonP1), or no modification (+GFP) or scrambled control (+shSCR) were incubated with methylcellulose for 14 days. The panel shows experimental design and graphical representation of the number of spheroids and representative images. % area of spheroids was measured. * **P < 0.005, compared to +GFP or +shSCR, using ANOVA. Error bars indicate the standard deviations of three biological replicates. n = 4 technical replicates from 3 biological replicates for each group.

We genetically manipulated LonP1 expression in the HOG astrocytoma cells and subjected them to BT317 treatment in a dose dependent manner. Cell viability was measured after 5 days. We first validated the LONP1 expression in the cells transduced for the gain (overexpression) and loss (knockdown) of LonP1 expression through immunoblotting (Fig. 5A). We found that vector control transfected cells i.e., +GFP with baseline LonP1 expression showed a moderate loss of cell viability with increasing concentrations of BT317. In contrast, the cells overexpressing LonP1 lost sensitivity to BT317 at even extremely high doses of 500 μM, making them ~15 fold less sensitive than the controls (Fig. 5B). In contrast, BT317 treated cells expressing scramble control shRNA showed similar moderate decrease in viability under gradient BT317 concentration, whereas cells expressing LonP1 shRNA with the LonP1 knockdown were significantly sensitive to gradient drug concentration of BT317 making them ~4 fold more sensitive than the control cells (Fig. 5B).

Similar with the previously published data in multiple myeloma [27], we also found that overexpression of the mitochondrial matrix protease LonP1 reduces the efficacy of the selective proteasome inhibitors CFZ in astrocytoma cells. Our viability assay on LonP1 over-expressed cells with IC_50_ = ~77 nM clearly showed approximately 12-fold less sensitivity to CFZ than the control cells with an IC_50_ = 6.5 nM. Whereas cells with LonP1 knockdown showed an IC_50_ = 0.6 nM thus increasing the cells sensitivity to CFZ by 10-fold when compared to scrambled control with an IC_50_ = 6.1 nM (Fig. 5C). These findings are confirmation that LonP1 overexpression derives resistance to proteasome inhibitors in astrocytoma cell, similar with the findings reported previously in multiple myeloma [27]. We further evaluated the apoptotic effect on the viability of these HOG overexpressing and knockdown LonP1 cells to understand the potential of CDDO-ME alone and in combination with 5 nM CFZ. As expected, LonP1 overexpression and knockdown regulates the sensitivity of HOG cells to CDDO-ME. LonP1 overexpressing cells shows two-fold less sensitivity with an IC_50_ value of ~820 nM to CDDO-ME compared to the GFP control cells with an IC_50_ value of ~430 nM. Surprisingly, 5 nM of CFZ sensitizes the LonP1 overexpressing cells to CDDO-ME (with an IC_50_ value of ~410 nM) to the extent that they become as sensitive as GFP cells (with an IC_50_ value of 430~ nM) whereas 5 nM of CFZ further sensitizes GFP control cells by further bringing down IC_50_ value to ~ 142 nM (Fig. 5C). Likewise, knocking down LonP1 sensitizes shLONP1 cells 3-fold (with an IC_50_ value of ~130 nM) to CDDO−ME compared to Scrambled control (SCR) (with an IC_50_ value of ~456 nM). Again, 5 nM of CFZ sensitizes further brings down the sensitivity of SCR control cells to CDDO-ME (with an IC_50_ value of ~137 nM) to the level of LonP1 knockdown in shLonP1 cells (with an IC_50_ value of ~130 nM), whereas 5 nM of CFZ sensitizes shLonP1 cells to an IC_50_ value of ~23 nM, which makes them 6-fold more sensitive to CDDO-ME (Fig. 5C). These results together clearly show that there is a direct relation between LonP1 and proteasome activity [27] and knocking down or overexpressing LonP1 regulates the sensitivity of cells to proteasome inhibitors.

Next, we evaluated apoptotic induction through ROS accumulation in our paired LonP1 overexpression and LonP1 knock-down HOG cells. BT317 treatment significantly induces ROS accumulation, and the combination of BT317 + TMZ showed a statistically significant increase in ROS accumulation as compared to BT317 alone, whereas TMZ alone has a relatively low ROS induction at 8 h incubation (Fig. 5D). We also found that LonP1 overexpression decreases ROS accumulation in cells compared to their respective control, and the combination treatment of BT317 + TMZ in the LonP1 overexpressing cells did not induce ROS even to reach baseline control transfected(+GFP) cells. In contrast, the knockdown of LonP1 significantly sensitized HOG to BT317 treatment alone and in combination with TMZ. These findings suggest that LonP1 provides increased tolerance to oxidative stress by decreasing the free radical accumulation in astrocytoma cells.

To further validate the functional significance of LonP1 expression in astrocytomas, we performed a spheroid forming assay in the HOG-LonP1 overexpressing cells and HOG-LonP1 downregulated cells, and the corresponding controls. Our results showed that LonP1 overexpression significantly increased spheroid formation. In contrast, LonP1 knock-down reduced spheroid formation (Fig. 5E). If gaining LonP1 expression allows astrocytoma cells to tolerate isolation stress in anchorage-independent conditions as demonstrated here through spheroid forming ability, then targeting this dependency may provide an opportunity to prevent these astrocytomas from evading the stresses encountered during cancer progression, aggressive local invasion and drug resistance. Accordingly, genetic inhibition of LonP1 decreased the levels stress tolerant phenotype in LonP1 knockdown astrocytomas as shown with the decreased spheroid forming ability (Fig. 5E).

### 3.6. BT317 exhibits low systemic toxicity, crosses the blood brain barrier (BBB), has tumor-specific activity, and increases survival in astrocytoma orthotopic mouse models

Development of proteasome inhibitors for the treatment of GBM has been limited by either poor BBB penetrance (i.e., BTZ) [24], central nervous system toxicity (i.e., MRZ=marizomib, confusion, ataxia) [44], or peripheral nervous system toxicity (BTZ, peripheral neuropathy) [45]. The development of these toxicities is directly related to the level of proteasome inhibition in the blood and in the normal brain [46].

Prior to evaluating efficacy, we established the maximum tolerated dose (MTD) of BT317. Using previously established methodology [37], we observed the clinical score following treatment (n = 2) with iterative 50 % dose escalation until we determined the MTD to be > 180 mg/kg (data not shown). We repeated a continuous dose series over 10 days with 100 mg/kg BT317 every other day or daily. There was no noticeable drop in weight or any observable clinical signs (Supplementary Fig. S6A). Next, we administered 100 mg/kg BT317, 25 mg/kg TMZ, or 100 mg/kg BT317 + 50 mg/kg TMZ daily for 10 days. A temporary drop in weight and minor clinical signs were observed following the first 2 doses with 100 mg/kg BT317 and 50 mg/kg TMZ; however, animal weights normalized by day 4 and no further clinical signs were observed. Liver toxicity was evaluated for gross morphological differences and vein diameter following 100 mg/kg BT317 administered daily for 10 days total. No visible signs of liver toxicity were detected with BT317 alone treatment, and a mild level of histological changes was noted when BT317 was administered in combination with TMZ (Supplementary Fig. S6A).

**Fig. 6.**
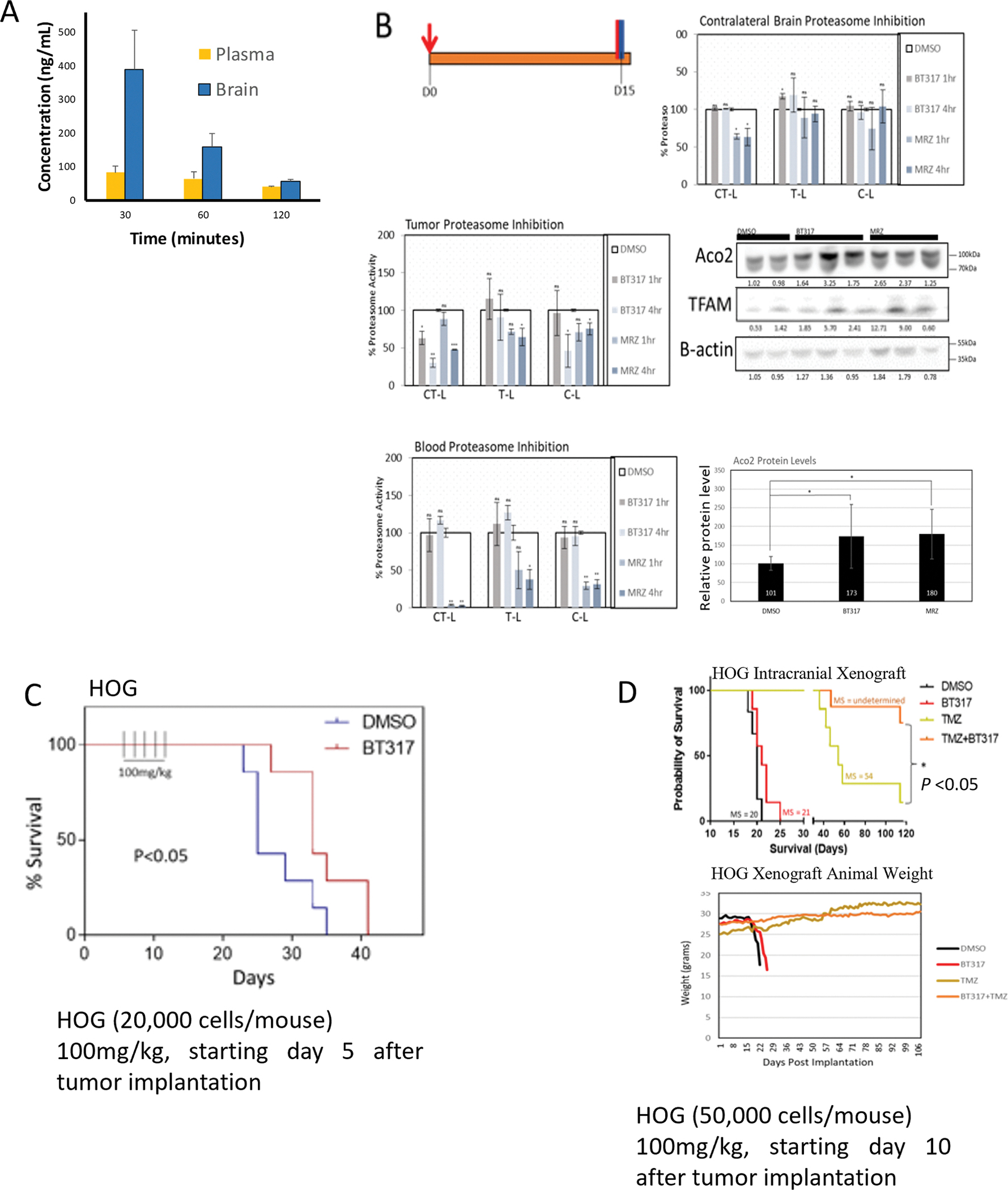
The Addition of BT317 to Temozolomide Significantly Improves Survival in Astrocytoma Orthotopic Xenograft Model. A. B6.129S7-Rag1^tm1Mom^/J mice received 3 mg/kg BT317 i.p. Blood and brain samples were harvested and analyzed via LC-MS for BT317 compound permeability levels. B. *In vivo* site-specific activity of BT317 was determined using B6.129S7-Rag1^tm1Mom^/J mice. HOG cells were intracranial implanted for 15 days, single dose of BT317 (100 mg/kg), Marizomib (50 μg/kg) or DMSO (w/v) were injected i.p. 1 h and 4 h prior to euthanasia. Proteasome activities were measured in the blood, healthy brain, and intracranial tumor mass. Protein levels of Aco2 and TFAM were measured in tumor tissue. Error bars indicate the standard deviations of six biological replicates (n = 6). Statistical significance was determined by 2-way ANOVA. * P < 0.05, * *P < 0.01, * **P < 0.001; n.s., not significant. C. B6.129S7-Rag1^tm1Mom^/J mice were intracranially implanted with 20,000 HOG cells/mouse. 100 mg/kg BT317 was injected i.p. every other day for 5 total doses starting on day 5 after intracranial implantation. Significance was measured n = 6 per endpoint). (BT317, n = 6; DMSO, n = 6 per endpoint). D. B6.129S7-Rag1^tm1Mom^/J mice were intracranially implanted with 50,000 HOG cells/mouse. 10 days after intracranial implantation, 100 mg/kg BT317 ± 100 mg/kg TMZ was injected i.p. daily for 5 days. The Log-rank method was applied to assess survival advantage in the intracranial xenograft model. Average weights of animals were measured following intracranial implantation of HOG cells. (BT317, n = 6; DMSO n = 6, TMZ n = 10, BT317 +TMZ n = 8 per endpoint).

From our initial modeling studies, we found all our synthetized compounds (see Fig. 2) have a logP value between 1.8 and 3.2 and our lead molecule BT317 logP is 2.66 (which is proximal range to cross BBB). To assess BBB penetrance, we engaged Cyprotex (https://www.cyprotex.com/). In short, B6.129S7-Rag1^tm1Mom^/J mice (n = 9, Jackson Laboratory) received a single injection of BT317 (3 mg/kg, i.p.). The animals were euthanized 30, 60, and 120 min post-BT317 injection (n = 3 per endpoint). Brain and plasma samples were collected, and BT317 (ng/mL) levels were quantified using mass spectrometry (Fig. 6A). BT317 levels reached ~390 ng/mL in the brain at 30 min and decreased to ~55 ng/mL at 120 min post-injection. At all measured endpoints, levels in the brain were significantly higher than those in the plasma.

To evaluate the target specific activity of BT317, we performed an intracranial implantation of the HOG line and 15 days post-implantation administered a single i.p. dose of 100 mg/kg BT317 (Fig. 6B). The animals were euthanized 1 h and 4 h post-injection (n = 6 per endpoint), and the proteasome activity was analyzed in the blood, normal brain, and intracranial tumor mass. BT317 showed ~70 % and 60 % inhibition of CT-L and C-L activity in the tumor, respectively, compared with ~50 % and 25 % for MRZ at 4 h. Only MRZ showed CT-L inhibition in the normal healthy brain with a ~40 % reduction at 1 h - 4 h. Furthermore, MRZ also inhibited CT-L, T-L, and C-L in the blood by ~90 %, 35–45 %, and 30 %, respectively. BT317 did not inhibit proteasome activity in the blood. Both BT317 and MRZ showed accumulation of Aco2 and TFAM in the tumor at 4 h with ~60–70 % increase (Fig. 6B).

Next, we assessed BT317 anti-tumor efficacy in the B6.129S7-Rag1^tm1Mom^/J mice KO mice intracranially implanted with the HOG tumor cells. We have used two experimental conditions, the first representing an early, small tumor model (20,000 cells/mouse with the experimental treatment started 5 days post-tumor implantation) and a second of a late, large intracranial tumor model (50,000 cells/mouse with the experimental treatments started ten days post-tumor implantation) Fig. 6C. Our data show that BT317 was effective in the small tumor model with the earlier dosing regimen (5 doses of 100 mg/kg BT317 every other day, starting on day 5) and significantly increased median survival from 25 to 33 days as compared to controls (p < 0.05).

In the late tumor model, a total of 5 doses were administered at 100 mg/kg BT317 ± 50 mg/kg TMZ daily, starting 10 days after intracranial xenograft implantation and continuing for a total of 5 days. Our results showed that BT317 alone improved the median survival by 1 days (p < 0.05) in the HOG line whereas, the combination of BT317 and TMZ significantly improved overall median survival (p < 0.01) as combination cohort median survival was undetermined by the end of the study (Fig. 6C., medium survival is 54 days for TMZ treated group, and over 120 days for the combination group, with 75 % of the animals alive 120 days after tumor implantation).

Together, here we found that BT317 is well tolerated, with good BBB permeability, on target tumor accumulation, showing little to no off-target proteasome inhibition. Further BT317 alone can control tumor growth for small tumors and can effectively be combined with TMZ to prolong median survival in large tumor orthotopic xenograft mouse models.

## 4. Discussion and conclusions

Potent therapeutics to control the frequent relapse and therapy resistance in malignant astrocytomas are lacking. Previously, we demonstrated that the LonP1 inhibitor, CC4, was effective against established glioma lines [19,47] and that proteasome inhibition with marizomib is effective in *in vitro* and *in vivo* glioma models, though its clinical use was limited by significant CNS toxicity [48,49]. Here we have reported the designing of the novel dual LonP1 and CT-L proteasome inhibitor, BT317, which has specific activity and limited toxicity with enhanced BBB permeability *in vivo,* on-target effects compared with currently available proteasome inhibitors.

For our rational design and SAR modeling, we used customized computational protein structure modeling programs, as well as standard programs, to identify lead compounds that could be derived from CC4 to improve solubility and BBB permeability while reducing toxicity. For compound design, we used the standard Lipinski rule of 5 with the following considerations: (1) pharmacological or biological activity (i.e., more sp3 carbon atoms), (2) ease of synthesis, and (3) moderate compound complexity to minimize toxicity and off-target effects [50].

Our lead molecule, BT317, exhibited dual inhibition of LonP1 protease activity and CT-L proteasomal activity, on-target LonP1 inhibition, BBB permeability, low animal toxicity, and prolonged survival with and without the standard-of-care (TMZ) in malignant astrocytoma intracranial xenograft models. BT317 accumulated at higher levels in the brain than in the plasma at 30 min post-administration. Its potent dual inhibitory activity can be compared with that of BTZ, a potent proteasome inhibitor that also inhibits LonP1 protease activity. However, BTZ has limited penetrance into the brain, and a phase II clinical trial of BTZ and bevacizumab in recurrent GBM was hindered by dose-limiting peripheral neuropathy associated with BTZ [51]. Marizomib has also shown improved survival in orthotopic GBM models; however, it has exhibited underlying issues with significant CNS toxicity (confusion, ataxia, fatigue) [52] in phase 2 studies and did not improve survival in a phase 3 randomized clinical trial [44]. This CNS toxicity is attributed to the high rate of marizomib-treated patients that have neurologic (67 %) and psychiatric (52 %) adverse events [48,49]. Additionally, the high MTD and specific tumor activity of BT317 further demonstrates its potential as a less toxic dual LonP1 and proteasomal inhibitor for astrocytomas treatment.

GBM is characterized by mitochondrial dysfunction, including metabolic shifts towards aerobic glycolysis, elevated ROS generation, and sensitivity to metabolic stress [19]. Aco2 participates in the tricarboxylic acid cycle (TCA) by converting citrate to isocitrate; however, its involvement in malignant astrocytoma metabolism is unclear. Recent studies have shown that Aco2 levels are decreased in breast cancer cell lines and patient-derived tumor biopsies, and Aco2 overexpression impairs breast cancer cell proliferation and mitigates the Warburg effect by redirecting pyruvate to the mitochondria [53]. LonP1 protease degrades Aco2 and prevents its accumulation [38]. Our findings revealed pronounced Aco2 accumulation in stable GBM lines and patient derived GSCs following BT317 exposure Fig. 3C. Notably, BT317 induced Aco2 accumulation at 10 μM BT317, which is lower than the IC_50_ = 31 μM for LonP1 protease inhibition [30,38]. BT317 also increased Aco2 levels in an intracranial xenograft model. Further investigation of the role of Aco2 accumulation in malignant astrocytoma metabolism is warranted.

An important finding of our study is that the combination treatment of BT317 + TMZ is more effective than either BT317 or TMZ alone in established and patient derived astrocytoma lines independent of their genetic profile, including IDH1/2 mutant/wildtype, ATRX (retention/loss), and p53 (mutant/wildtype). We previously found that LonP1 is increased in aggressive and malignant subtypes of astrocytomas [19]. LonP1 is directly involved in imparting tolerance under isolation stress in astrocytomas as observed in colony formation assay (Fig. 5E) which is an established mechanism of aggressive recurrence and drug resistance. The normal human fibroblast cell lines, HPF242 and MCF-10A, were less sensitive to BT317 treatment than the astrocytoma lines (Supplementary Fig. 3A).

This study further validated that LonP1 is required to drive resistance (gain of function study) and sufficient to drive sensitivity (loss of function study) to BT317 when evaluated using cell viability and colony formation assays (Fig. 5B and E). Targeting LonP1 by BT317 increases cellular ROS levels inducing apoptosis in both established and patient derived malignant astrocytomas Fig. 5D. We further found that overexpression of LonP1 reduces the efficacy of proteasome inhibitor, CFZ, and likewise knocking down LonP1 sensitizes astrocytoma cells to CFZ, thus showing the role of LonP1 in increasing treatment resistance to proteasome inhibitors, Fig. 5C. The protective effect of LonP1 also occurs when using drugs like CFZ that do not block its protease activity, suggesting that LonP1 may compensate for loss of proteasome activity.

Targeting the GSC population is critical for overcoming glioma treatment resistance. Proteasome activity also plays a key role in cancer treatment resistance [22,23]. Interestingly, gliomas have elevated CT-L proteasome activity and, generally, an increase in this activity serves as a compensatory response to pro-oxidative treatment [54]. Our *in vitro* findings suggest that patient-derived GSC lines are particularly sensitive to LonP1 inhibition, and that dual-combination approach with BT317 plus TMZ may be a potential therapeutic strategy in malignant astrocytoma. Additional studies are vital to further explore the role of LonP1 and CT-L proteasome activity in malignant glioma metabolism and whether dual inhibition could be useful for treating recurrent malignant astrocytoma based on patient-specific genetic determinants. Future work will also seek to understand the best route of administration, while also creating BT317 analogs that incorporate nanoparticles and other moieties to optimize on-site and on-target activity to further the development of new therapeutic options for malignant astrocytoma patients.

## Supplementary Material

Supplementary Materials

plementary Materials

## Data Availability

Data will be made available on request.

## Data access statement

Research data supporting this publication are as presented in the manuscript.

## Appendix A. Supporting information

Supplementary data associated with this article can be found in the online version at doi:10.1016/j.phrs.2025.107697.
