# Supplementary material for: Dual targeting of the mitochondrial Lon peptidase 1 and the chymotrypsin-like proteasome activity as a potential therapeutic strategy in malignant astrocytoma models
Source: Pharmacol Res. Author manuscript; Available in PMC 2026 Mar 2. (PMC12952243; doi:10.1016/j.phrs.2025.107697)
Supplement: Supplementary Materials [file NIHMS2137128-supplement-Supplementary_Materials.pptx]

## Slide 1
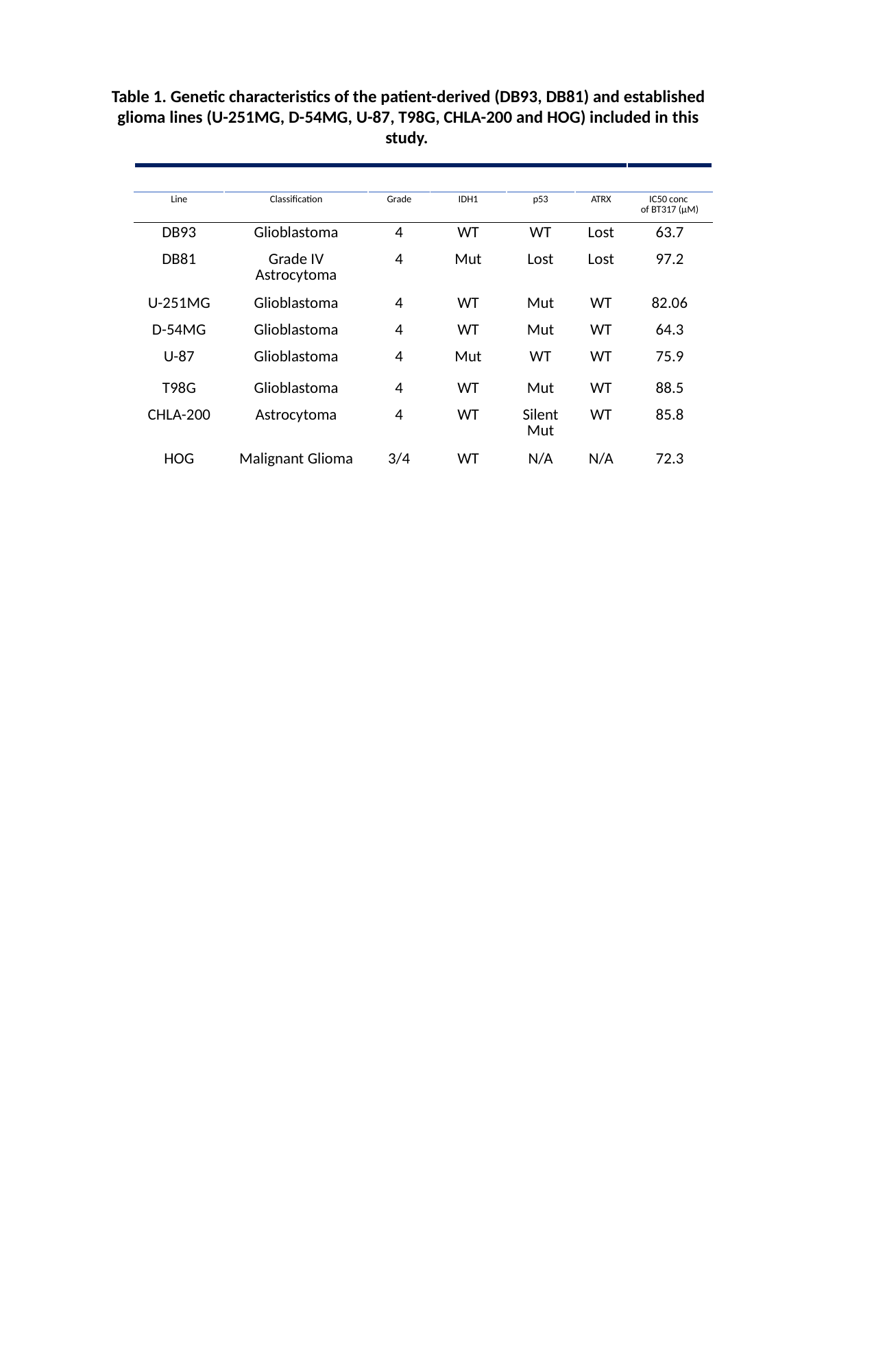

Table 1. Genetic characteristics of the patient-derived (DB93, DB81) and established glioma lines (U-251MG, D-54MG, U-87, T98G, CHLA-200 and HOG) included in this study.
| | | | | | | |
| --- | --- | --- | --- | --- | --- | --- |
| Line | Classification | Grade | IDH1 | p53 | ATRX | IC50 conc of BT317 (µM) |
| DB93 | Glioblastoma | 4 | WT | WT | Lost | 63.7 |
| DB81 | Grade IV Astrocytoma | 4 | Mut | Lost | Lost | 97.2 |
| U-251MG | Glioblastoma | 4 | WT | Mut | WT | 82.06 |
| D-54MG | Glioblastoma | 4 | WT | Mut | WT | 64.3 |
| U-87 | Glioblastoma | 4 | Mut | WT | WT | 75.9 |
| T98G | Glioblastoma | 4 | WT | Mut | WT | 88.5 |
| CHLA-200 | Astrocytoma | 4 | WT | Silent Mut | WT | 85.8 |
| HOG | Malignant Glioma | 3/4 | WT | N/A | N/A | 72.3 |

## Slide 2
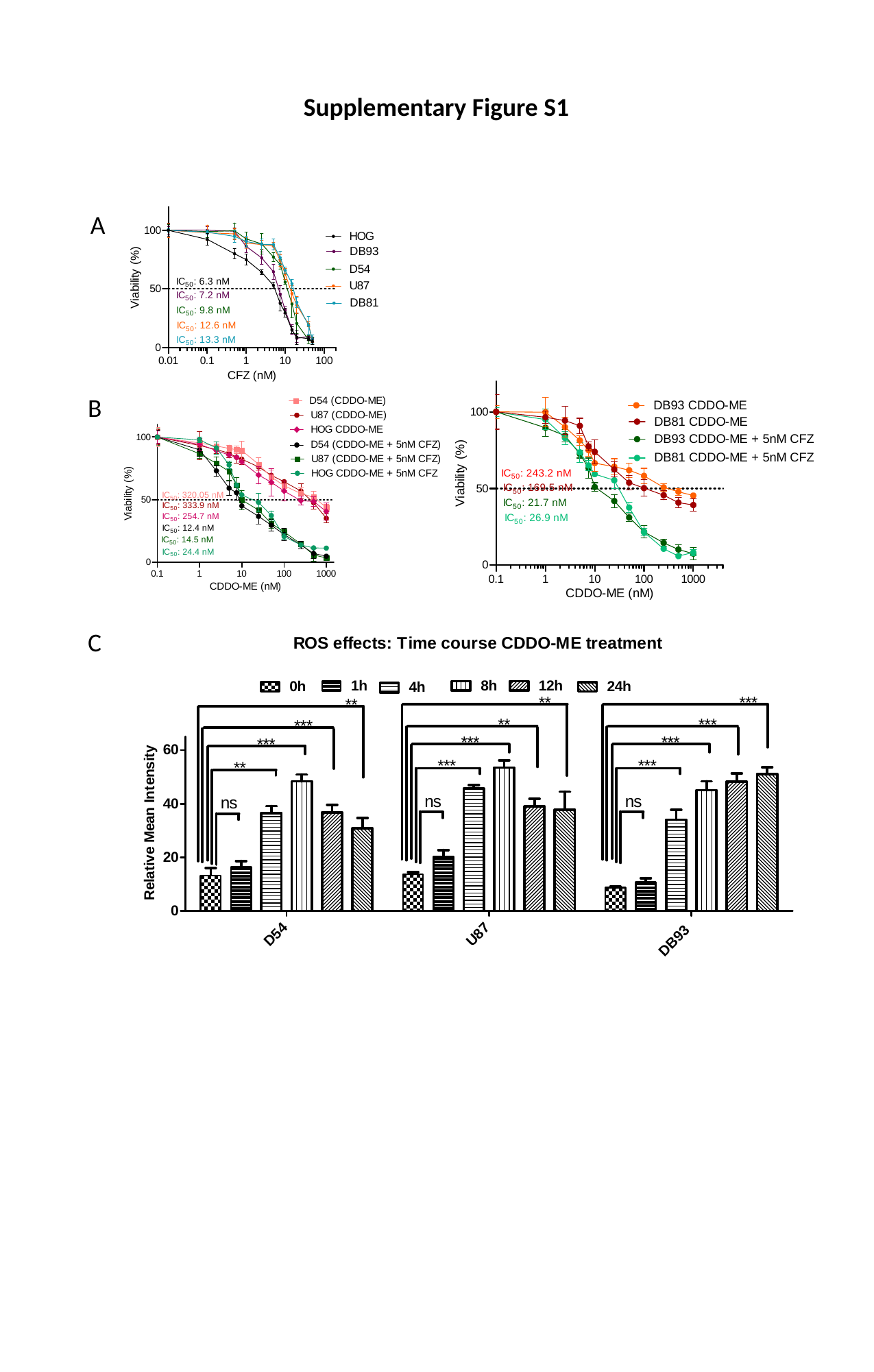

Supplementary Figure S1
A
B
C

## Slide 3
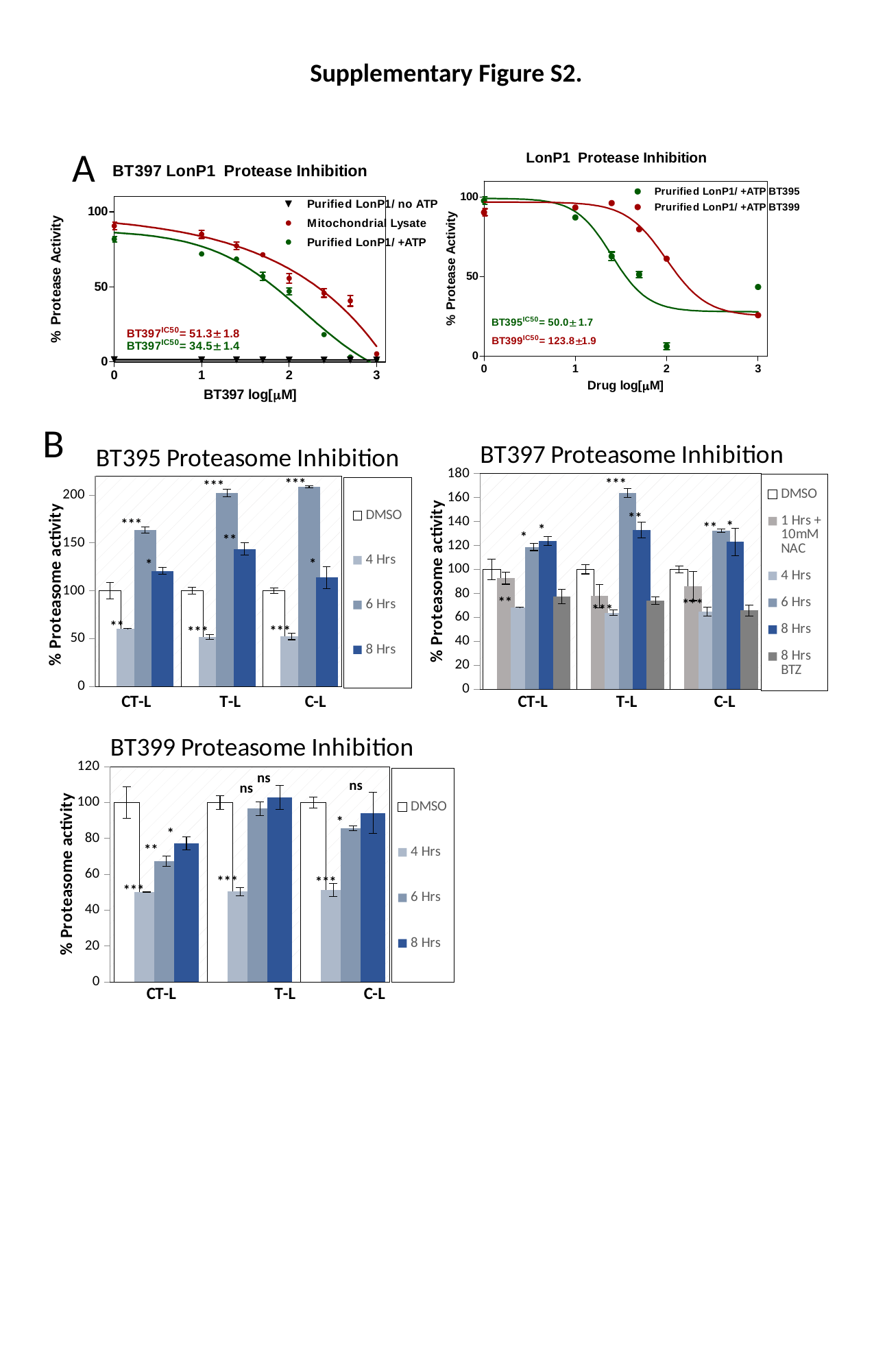

Supplementary Figure S2.
A
B
### Chart: BT397 Proteasome Inhibition
| Category | DMSO | 1 Hrs + 10mM NAC | 4 Hrs | 6 Hrs | 8 Hrs | 8 Hrs BTZ |
|---|---|---|---|---|---|---|
| CT-L | 100.0 | 92.66917293233082 | 68.47012475377544 | 118.7274290627687 | 123.61134995700775 | 77.48925193465176 |
| T-L | 100.0 | 77.67584097859327 | 63.86112572330354 | 163.73390557939913 | 132.8326180257511 | 73.92703862660943 |
| C-L | 100.0 | 86.20828307197425 | 64.74098182804447 | 132.18270571827057 | 122.90794979079497 | 65.62064156206416 |***
**
*
**
*
*
**
***
***
### Chart: BT395 Proteasome Inhibition
| Category | DMSO | 4 Hrs | 6 Hrs | 8 Hrs |
|---|---|---|---|---|
| CT-L | 100.0 | 60.60407091267235 | 163.6457437661221 | 120.63628546861565 |
| T-L | 100.0 | 51.65702261967386 | 202.46781115879827 | 143.77682403433477 |
| C-L | 100.0 | 52.40032546786005 | 208.64714086471412 | 113.7726638772664 |***
***
***
**
*
*
**
***
***
### Chart: BT399 Proteasome Inhibition
| Category | DMSO | 4 Hrs | 6 Hrs | 8 Hrs |
|---|---|---|---|---|
| CT-L | 100.0 | 50.09848982271832 | 67.34307824591573 | 77.33447979363714 |
| T-L | 100.0 | 50.34192530247238 | 96.56652360515021 | 102.78969957081544 |
| C-L | 100.0 | 51.152698671006235 | 85.56485355648536 | 94.21199442119944 |ns
ns
ns
*
*
**
***
***
***

## Slide 4
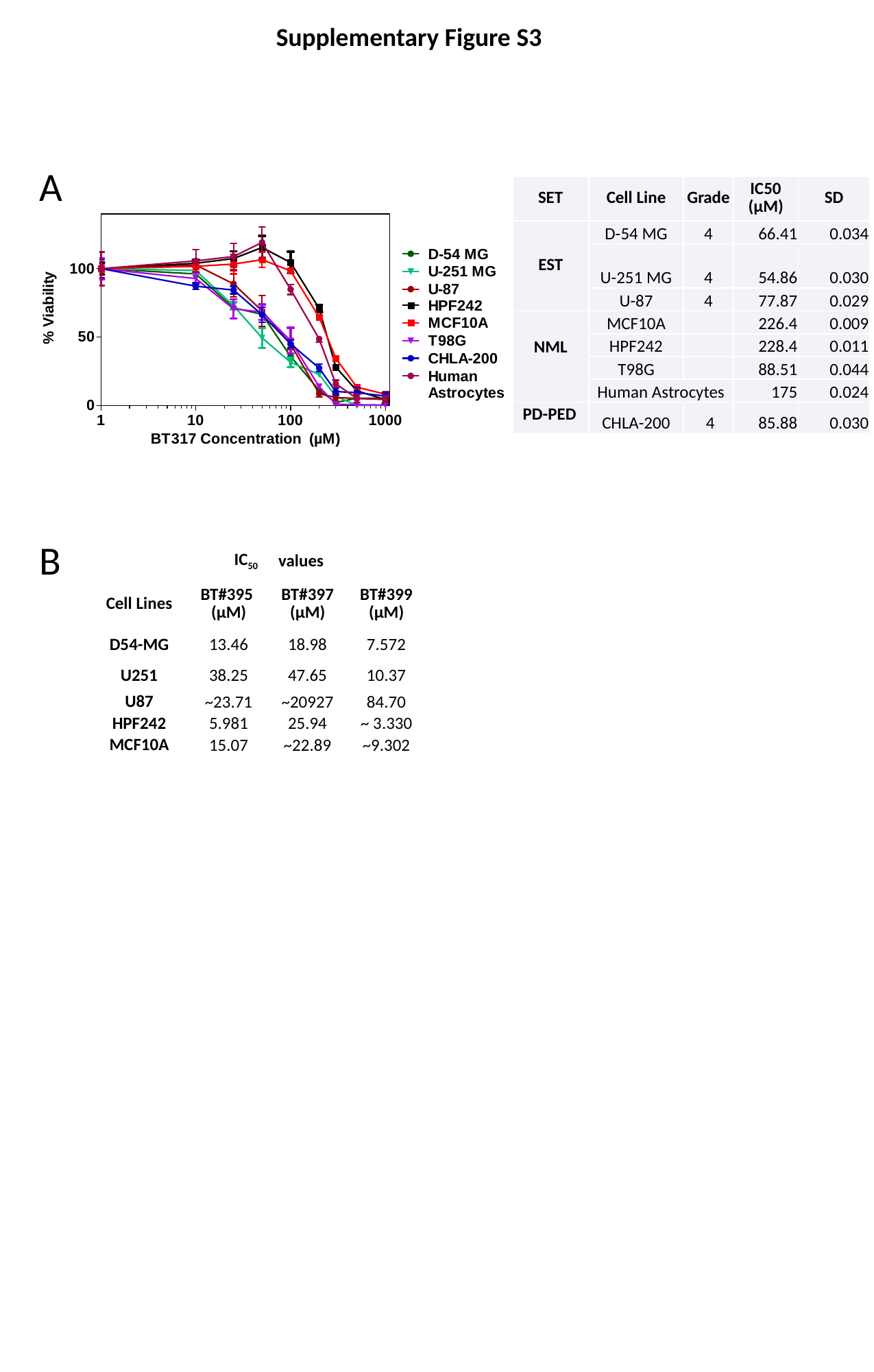

Supplementary Figure S3
A
| SET | Cell Line | Grade | IC50 (µM) | SD |
| --- | --- | --- | --- | --- |
| EST | D-54 MG | 4 | 66.41 | 0.034 |
| | U-251 MG | 4 | 54.86 | 0.030 |
| | U-87 | 4 | 77.87 | 0.029 |
| NML | MCF10A | | 226.4 | 0.009 |
| | HPF242 | | 228.4 | 0.011 |
| | T98G | | 88.51 | 0.044 |
| | Human Astrocytes | | 175 | 0.024 |
| PD-PED | CHLA-200 | 4 | 85.88 | 0.030 |
B
| | IC50 | values | |
| --- | --- | --- | --- |
| Cell Lines | BT#395 (µM) | BT#397 (µM) | BT#399 (µM) |
| D54-MG | 13.46 | 18.98 | 7.572 |
| U251 | 38.25 | 47.65 | 10.37 |
| U87 | ~23.71 | ~20927 | 84.70 |
| HPF242 | 5.981 | 25.94 | ~ 3.330 |
| MCF10A | 15.07 | ~22.89 | ~9.302 |

## Slide 5
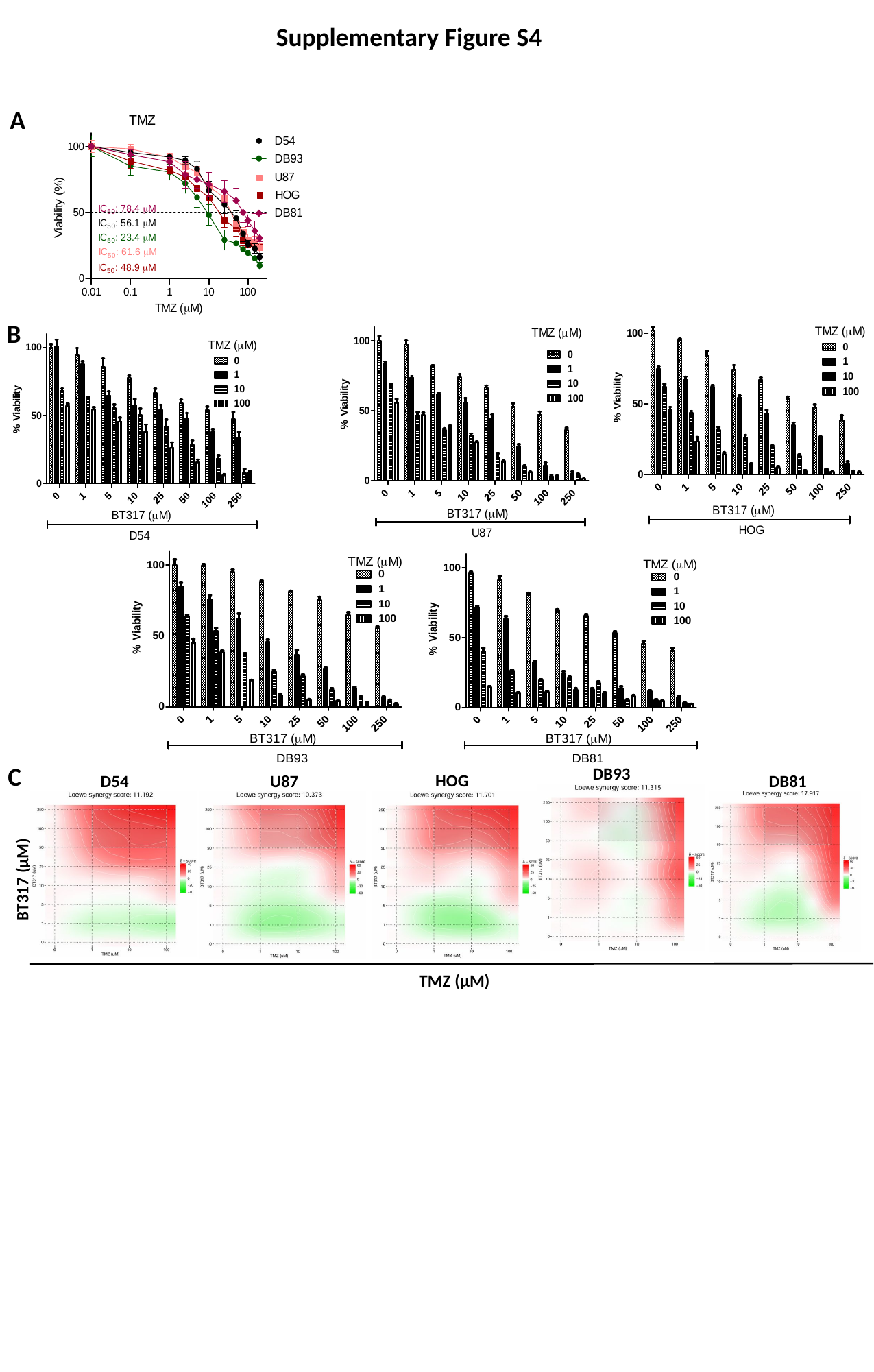

Supplementary Figure S4
A
B
C
DB93
HOG
D54
U87
BT317 (µM)
TMZ (µM)
DB81

## Slide 6
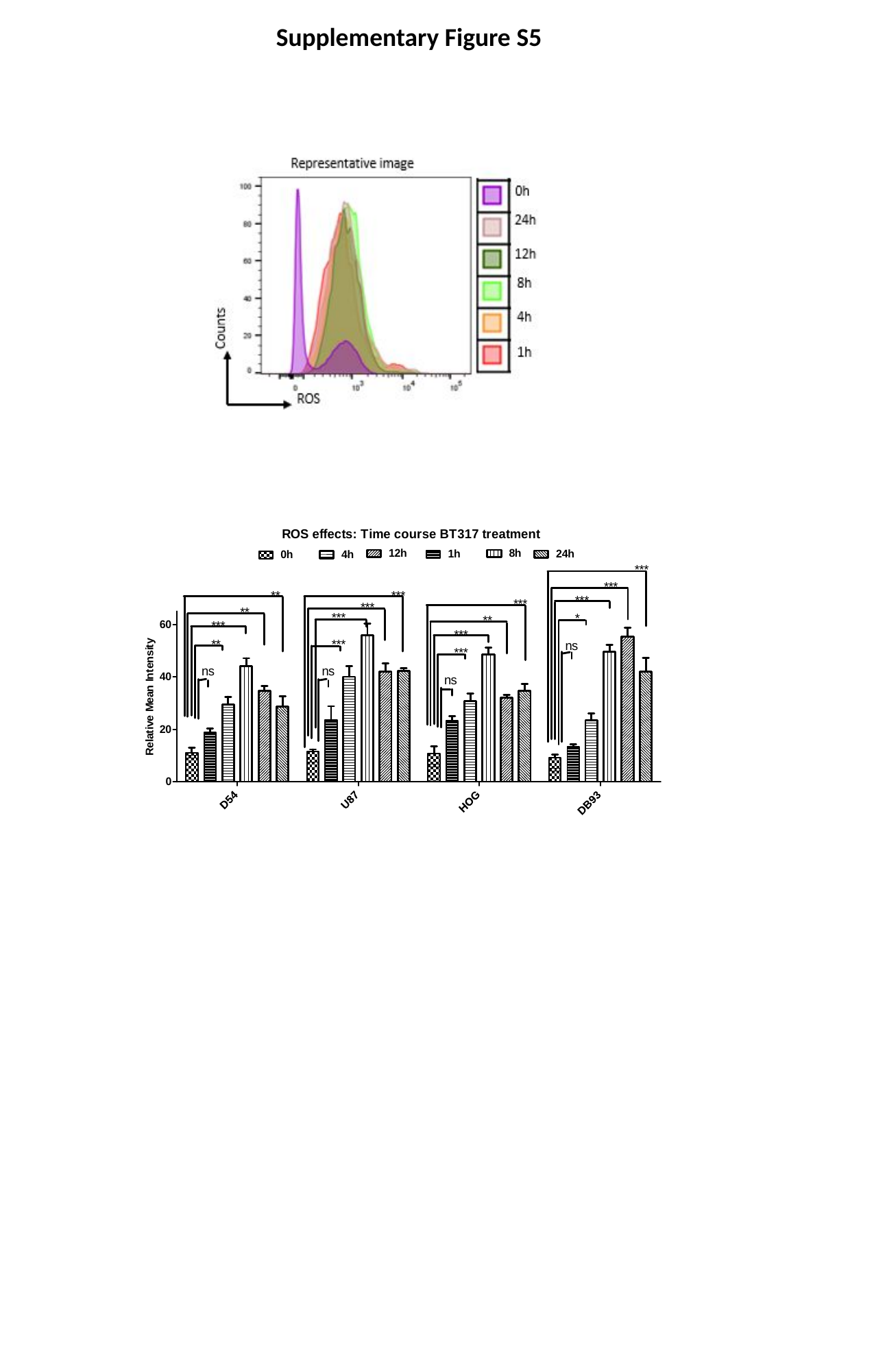

Supplementary Figure S5

## Slide 7
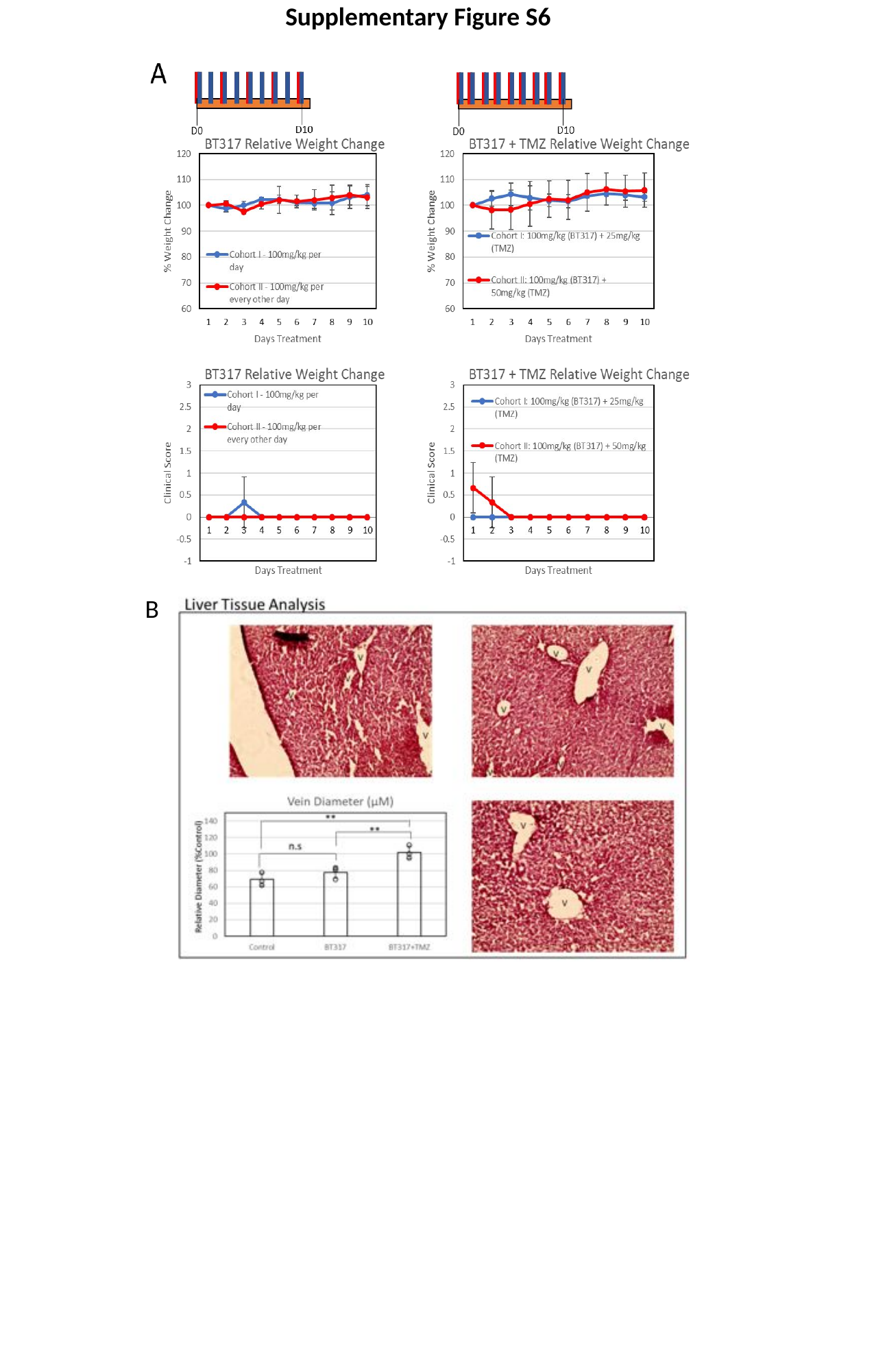

Supplementary Figure S6
B
