# Supplementary material for: Dual targeting of the mitochondrial Lon peptidase 1 and the chymotrypsin-like proteasome activity as a potential therapeutic strategy in malignant astrocytoma models
Source: Pharmacol Res. Author manuscript; Available in PMC 2026 Mar 2. (PMC12952243; doi:10.1016/j.phrs.2025.107697)
Supplement: plementary Materials [file NIHMS2137128-supplement-plementary_Materials.docx]

**Supplementary Table**

**Supplementary Table 1. Genetic characteristics of the patient-derived (DB93, DB81) and established glioma lines (U-251MG, D-54MG, U-87, T98G, CHLA-200 and HOG) included in this study.**

**Supplementary Figures Legends**

**Supplementary Figure S1. LonP1 Protease Inhibitor CDDO-Me and Chymotrypsin-like Proteasome Inhibitor CFZ have Greater Synergy in Combination in Malignant Astrocytoma Cell Lines. A.** D54-MG, U87, HOG, DB93, and DB81 astrocytoma cells were incubated with indicated concentration of CFZ, for 48h. Cell viability was detected at the end of incubation using XTT reagent. Error bars indicate the standard deviations of three biological replicates. n = 3 technical replicates from 3 biological replicates for each group. Statistical significance was determined by t-test. **B.** D54-MG, U87, HOG, DB93, and DB81 cells were incubated with the indicated concentrations of CDDO-ME with or without CFZ (5 nM) for 48 h. The cell viability was measured at the end of the incubation period using XTT reagent. Error bars indicate the standard deviations of three biological replicates. n = 3 technical replicates from 3 biological replicates for each group. **C.** Relative ROS generation was measured in D54-MG, U87, and DB93 cells after incubation for the indicated time with 200 nM CDDO-ME. The relative mean intensities of ROS accumulation were measured at the end of the incubation period. Error bars indicate the standard deviations of three biological replicates. n = 3 technical replicates from 3 biological replicates for each group.

**Supplementary Figure S2. BT395, BT397 and BT399 Show Limited Inhibition of LonP1 and Proteasome Activity. A.** LonP1 Protease was purified through antibody crosslinking and Immunoprecipitated from mitochondrial lysate of HOG cells. Gradient concentrations of BT397, BT395, and BT399 were added to purified LonP1 protease lysate or equivalent total mitochondrial lysate for BT397 and incubated for 1h at 37°C. At the end of incubation, fluorescent FITC-Casein substrate was added to measure the effect of BT397, BT395, and BT399 on enzyme activity. BT397, BT395, and BT399 were assessed for the inhibition of Lon protease activity in the purified LonP1 protease assay with IC_50s_ established at 34.5, 50.0, and 123.8 µM, whereas BT397 showed the inhibition of Lon protease activity with IC_50_ 51.3 µM in total mitochondrial lysate. Subtracting ATP from the reaction deactivates the overall LonP1 enzyme activity. The calculations are based on results from three independent experiments. **B.** 20 µM BT395, BT397, and BT399 were evaluated for proteasome inhibition at 1, 4, 6, 8 h in D54-MG lysates. BT395, BT397, and BT399 have variable levels of short-term proteasome inhibition. CT-L Chymotrypsin-like activity; T-L Trypsin-like activity; C-L Caspase-like activity. Statistical significance was determined by t-test. * P <0.05, **P <0.01, ***P <0.001; n.s., not significant.

**Supplementary Figure S3  Evaluation of Multiple Inhibitors Toxicity in Normal Cell Lines as Compared with Malignant Astrocytoma Lines. A.** Multiple cell lines were incubated with the indicated concentrations of BT317 for 120 h. The cell viability was measured at the end of the incubation period. Error bars indicate the standard deviations of three biological replicates. n = 3 technical replicates from 3 biological replicates for each group.  **B.** Multiple cell lines were incubated with varying concentrations of BT395, BT397, and BT399 for 5 days. Cell viability was detected at the end of incubation using XTT reagent. n = 3 technical replicates from 3 biological replicates for each group.

**Supplementary Figure S4. BT317 Shows Strong Synergy with TMZ in Malignant Astrocytoma Lines. A.** Relative sensitivity of D54-MG, U87, HOG, DB93 and DB81 cells to TMZ. The cells were incubated with the indicated concentrations of TMZ for 120 h. The cell viability was measured at the end of the incubation period. Error bars indicate the standard deviations of three biological replicates. n = 3 technical replicates from 3 biological replicates for each group. **B.** D54-MG, U87, HOG, DB93, and DB81 cells were incubated with the indicated concentrations of BT317 with or without indicated concentrations of TMZ for 120 h. The cell viability was measured using Annexin PI reagent at the end of the incubation period using flow cytometry. Error bars indicate the standard deviations of three biological replicates. n = 3 technical replicates from 3 biological replicates for each group. **C.** Synergy test between BT317 and TMZ was evaluated using BIG, with red representing significant synergy and green representing anergy. Malignant astrocytoma cells were incubated with the indicated concentrations of BT317 with or without TMZ (0, 1, 10, and 100 µM) for 120 h. The cell viability data from the flow cytometry was used to interpret synergy. n = 3 technical replicates from 3 biological replicates for each group. A synergy test between BT317 and TMZ combination was performed with this XTT data using the online tool [SynergyFinder - Documentation (fimm.fi)](https://synergyfinder.fimm.fi/synergy/synfin_docs/).

**Supplementary Figure S5**. **BT317 Induces ROS Production in Multiple Astrocytoma Cell Lines.** D54-MG, U87, HOG, and DB93 cell lines were incubated with 20 µM BT317 for 1, 4, 8, 12 and 24 h. ROS levels were detected at the end of incubation using the deep red oxidative stress reagent through flow cytometry. Relative mean intensities were calculated for each group. Error bars indicate the standard deviations of three biological replicates. n = 3 technical replicates from 3 biological replicates for each group. Statistical significance was determined by 2way ANOVA. * P <0.05, **P <0.01, ***P <0.001; n.s., not significant.

**Supplementary Figure S6. BT317 as a Single Agent and in Combination with TMZ is Well Tolerated in the Mouse Model, with no Weight Loss and Limited Liver Toxicity. A.** Maximum tolerated dose of BT317 was determined alone and in combination with TMZ. BT317 was administered intraperitoneally (i.p.) at 100mg/kg every other or daily for 10 total days, and 100mg/kg of BT317 in combination with 25 and 50 mg/kg TMZ were administered daily for 10 days. The percent weight change and the clinical scores were monitored daily for all animal groups. **B.** Liver toxicity was evaluated for gross morphological differences and vein diameter changes following 100mg/kg of BT317 administered alone and in combination with TMZ (50mg/kg) daily for 10 days. Statistical significance was determined by t-test. **P <0.01, n.s., not significant.

**Supplementary Methods**

*Synthesis of Small molecule BT317 and Related Compounds*: Synthesis of BT317 was initiated using compound D (methyl 6-chloro-2-oxo-2H-chromene-3-carboxylate) (Fig. 2C). Compound D was then synthesized from Compound A using the outlined process by first adding 5-chlorosalicyclaldehyde (A, 1.55 g, 10 mM) to CH3CN (5 mL) in a 35 mL reaction tube. Dimethylmalonate (B) (1.45 g, 11 mM) and ethyl piperdine-3-carboxylate (C) (15 mg, 10 mol%) in 15 mL of CH3CN were added. The resulting reaction mixture was stirred at room temperature for 24 h. After the evaporation of CH3CN, the crude reaction mixture was purified by silica gel chromatography to give a white solid D (1.6 g, 60%). Basic hydrolysis of compound D was performed to produce Compound E acid (6-chloro-2-oxo-2H-chromene-3-carboxylic acid). Acid synthesis was initiated by adding methyl 6-chloro-2-oxo-2H-chromene-3-carboxylate E (1.0 g, 4.2 mM) in ethanol to sodium hydroxide (10% w/v in 20 mL ethanol). The mixture was stirred under reflux for 24 h. After the completion of the reaction, the mixture was cooled to room temperature and diluted with 10% HCl. The solid precipitated out and was isolated and washed with water, yielding compound E (white solid, 80% yield). Using compound E, we further derivatized to amide (BT173) using a simple amide coupling reaction in the presence of coupling reagents. The 6-chloro-2-oxo-2H-chromene-3-carboxylic acid E (1.0 mM) was dissolved in dichloromethane (DCM) (3 mL) in a 35 mL reaction tube, then 4-hydroxy aniline (1.1 mM) and DCC (1.2 mM) were added. The resulting reaction mixture was stirred at room temperature for 24 h. The reaction was then quenched with a saturated aqueous NaOH solution and extracted with DCM (10 × 3 mL). The combined organic layers were dried over Na_2_SO_4_ and then filtered. After evaporation of the organic solvent, the residue was purified by silica gel chromatography to obtain BT317, 6-Chloro-N-(4-hydroxyphenyl)-2-oxo-2H-chromene-3-carboxamide (Fig. 2C; white solid, 78% yield). The reagents used for the synthesis were purchased from Fisher Scientific. The structure was confirmed by nuclear magnetic resonance (NMR) and proton, carbon, and mass spectrometry, and the purity of the compound was determined by HPLC. The lead compound, BT317, was evaluated using NMR as follows: 1H NMR (500 MHz, DMSO-d6) δ 10.44 (s, 1H), 9.43 (s, 1H), 8.87 (s, 1H), 8.16 (d, J = 2.5 Hz, 1H), 7.82 (dd, J = 8.8, 2.6 Hz, 1H), 7.61 (d, J = 8.9 Hz, 1H), 7.53 (d, J = 8.5 Hz, 2H), 6.78 (d, J = 8.4 Hz, 2H). 13C NMR (125 MHz, DMSO) δ 160.0, 158.8, 154.2, 152.4, 145.7, 134.2, 133.5, 129.5, 129.0, 121.6, 121.2, 119.9, 118.2, 115.3. HRMS (ESI) were calculated for C16H11ClNO4 (M.W. = 316.0371) and 316.0607 [M + H]+.

**Detailed Compound Synthesis**

**General Information**

All reagents were purchased from commercial sources and used without treatment, unless otherwise indicated. The products were purified by column chromatography over silica gel. ^1^H NMR and ^13^C NMR spectra were recorded at 25 °C at 600 MHz and 125 MHz, respectively, with TMS as internal standard. Abbreviations for signal coupling are as follows: s, singlet; d, doublet; t, triplet; q, quartet; m, multiplet; br, broad. Column chromatography was performed using SiO_2_ (0.060 – 0.2 mm, 70 - 230 mesh) from Alfa Aeser. All reagents were obtained from commercial sources. Mass spectra were recorded on Varian MS mass spectrometer.

**General procedure of synthesis of 2-oxo-2H-chromene:**

**methyl 6-chloro-2-oxo-2H-chromene-3-carboxylate (2)**. Procedure for synthesis of Methyl 6-chloro-2-oxo-2H-chromene-3-carboxylate: 5-chlorosalicyclaldehyde (1.55 g, 10 mmol) was added into CH_3_CN (5 mL) in a 35 mL reaction tube. To this dimethylmalonate (1.45 g, 11 mmol), ethyl piperdine-3-carboxylate (15 mg, 10 mol%) in 15 mL CH_3_CN were added. The resultant reaction mixture was stirred at room temperature for 24 hrs. After evaporation of the acetonitrile, the crude reaction reaction mixture was purified by a silica gel chromatography to give a white solid **BT301** (1.6 g, 60%). ^1^H NMR (DMSO, 600 MHz) δ 8.71 (s, 1H), 8.01 (s, 1H), 7.75 (d, J = 9.6 Hz, 1H), 7.45 (d, J = 9.3 Hz, 1H), 3.82 (s, 3H) ppm; ^13^C NMR (DMSO, 75 MHz) δ 163.2, 161.9, 148.5, 147.7, 134.9, 134.3, 129.9, 128.4, 118.9, 118.4, 53.1 ppm. HRMS (EI) Calcd. for C_11_H_7_ClO_4_ [M+H]^+^ requires 239.0111, found 239.0117.

**6-chloro-2-oxo-2H-chromene-3-carboxylic acid (3)**. Procedure for synthesis of 6-chloro-2-oxo-2H-chromene-3-carboxylic acid: to a solution of methyl 6-chloro-2-oxo-2H-chromene-3-carboxylate (1.0 g, 4.2 mmmol) in ethanol was added sodium hydroxide (10% w/v in 20 mL ethanol). This mixture was stirred under reflux conditions for 24 hrs. Further after completion of the reaction, the reaction mixture was cooled down room temperature and diluted with 10% HCl. The solid precipitaed out was isolated and washed with water to afford the product as a white solid BT302(0.75 g, 80%). ^1^H NMR (DMSO, 300 MHz) δ 13.38 (s, 1H), 8.68 (s, 1H), 8.02 (s, 1H), 7.74 (d, J = 9 Hz, 1H), 7.46 (d, J = 9 Hz, 1H) ppm; ^13^C NMR (DMSO, 75 MHz) δ 164.1, 156.5, 153.48, 128.8, 119.9, 119.7, 118.5, 111.48, 102.19 ppm. HRMS (EI) Calcd. for C_10_H_5_ClO_4_ [M+H]^+^ requires 224.9955, found 224.9948.

**General procedure of synthesis of 2-oxo-2H-chromene amide derivatives**:

The 6-chloro-2-oxo-2H-chromene-3-carboxylic acid (1.0 mmol) was dissolved in DCM (3 mL) in a 35 mL reaction tube. To this solution was added 4-amino phenyl boronic acid pinacol ester (1.1 mmol) and DCC (1.2 mmol). The resultant reaction mixture was stirred at room temperature for 24 hrs. After completion of the reaction, the reaction was then quenched by saturated aq. NaOH solution, extracted with DCM (10 × 3 mL). The combined organic layers were dried over Na_2_SO_4_ and filtered. After evaporation of the organic solvent, the residue was purified by a silica gel chromatography to provide the product.

**6-chloro-N-(4-methyl-3-(trifluoro-l4-boraneyl)phenyl)-2-oxo-2H-chromene-3-carboxamide, potassium salt (BT-300)**. A yellow solid (55 mg, 77%). m.p. 118-120 ^o^C. ^1^H NMR (DMSO, 600 MHz) δ 10.39 (s, 1H), 8.85 (d, *J* = 1.2 Hz, 1H), 8.14 (d, *J* = 2.4 Hz, 1H), 7.77 (dd, *J_1,2_* = 9.0, 3.0 Hz, 1H), 7.58-7.56 (m, 2H), 7.27 (d, J = 2.4 Hz, 1H), 6.88 (d, *J* = 7.8 Hz, 1H), 2.26 (s, 3H); ^13^C NMR (CDCl_3_, 75 MHz) δ 160.8, 159.0, 158.9, 152.9, 146.1, 137.7, 134.0, 133.9, 133.8, 129.5, 129.3, 129.0, 123.9, 123.8, 121.8, 120.5, 118.7, 117.2, 117.1. HRMS (EI) Calcd. for C_17_H_11_BClF_3_KNO_3_ [M+H]^+^ requires 442.0007, found 442.1383.

**6-chloro-2-oxo-N-(4-(4,4,5,5-tetramethyl-1,3,2-dioxaborolan-2-yl)phenyl)-2H-chromene-3-carboxamide** **(BT-304)**. A yellow solid (95 mg, 44%). ^1^H NMR (CDCl_3_, 600 MHz) δ 10.71 (s, 1H), 8.83 (s, 1H), 8.14 (d, J = 2.4 Hz, 1H), 7.80 (dd, *J_1,2_* = 9.0, 2.4 Hz, 2H), 7.72 (d, J = 8.4 Hz, 2H), 7.68 (d, *J* = 8.4 Hz, 2H), 7.58 (d, J = 9 Hz, 3H), 1.28 (s, 12H) ppm; ^13^C NMR (CDCl_3_, 150 MHz) δ 160.3, 155.8, 152.9, 146.4, 141.0, 135.9, 121.8, 120.2, 119.4, 118.7, 107.2, 84.0, 25.1 ppm. HRMS (EI) Calcd. for C_22_H_21_BClNO_5_ [M+Na]^+^ requires 426.1280, found 426.1246.

**6-chloro-N-(4-methyl-3-(4,4,5,5-tetramethyl-1,3,2-dioxaborolan-2-yl)phenyl)-2-oxo-2H-chromene-3-carboxamide** **(BT-305))**. A yellow solid (276 mg, 63%). m.p. 118-120 ^o^C. ^1^H NMR (CDCl_3_, 600 MHz) δ 10.65 (s, 1H), 8.93 (s, 1H), 7.95 (dd, *J_1,2_* = 7.8, 2.4 Hz, 1H), 8.82 (d, J = 2.4 Hz, 1H), 7.69 (d, *J* = 2.4 Hz, 1H), 7.63 (dd, *J_1,2_* = 9.0, 2.4 Hz, 1H), 7.39 (d, *J*  = 9.0 Hz, 1H), 7.19 (d, *J* = 8.4 Hz, 1H), 2.5 (s, 3H), 1.35 (s, 12H); ^13^C NMR (CDCl_3_, 175 MHz) δ 160.3, 152.9, 146.2, 140.5, 135.3, 134.0, 129.5, 129.4, 127.4, 127.3, 123.0, 122.9, 121.9, 120.3, 118.7 ppm. HRMS (EI) Calcd. for C_23_H_23_BClNO5 [M+Na]^+^ requires 439.1358, found 439.1433.

**6-chloro-2-oxo-N-(4-(4,4,5,5-tetramethyl-1,3,2-dioxaborolan-2-yl)benzyl)-2H-chromene-3-carboxamide (BT-307)**. A yelow solid (197 mg, 45%). ^1^H NMR (CDCl_3_, 600 MHz) δ 9.11 (t, J = 6 Hz, 1H), 8.82 (s, 1H), 8.11 (d, *J* = 3 Hz, 1H), 7.75 (dd, *J_1,2_* = 8.4, 2.4 Hz, 1H), 7.62 (d, *J* = 7.2 Hz, 2H), 7.53 (d, J = 9.0 Hz, 1H), 7.33 (d, *J*  = 7.8 Hz, 2H), 4.54 (d, *J* = 6.0 Hz, 2H), 1.26 (s, 12H); ^13^C NMR (CDCl_3_, 75 MHz) δ 161.5, 160.3, 152.9, 146.7, 142.8, 135.0, 133.9, 129.5, 129.5, 127.2, 120.7, 120.3, 118.6, 84.0, 53.7, 25.1. HRMS (EI) Calcd. for C_23_H_23_BClNO_5_ [M]^+^ requires 439.1358, found 437.3827.

**6-chloro-N-(2-nitro-4-(4,4,5,5-tetramethyl-1,3,2-dioxaborolan-2-yl)phenyl)-2-oxo-2H-chromene-3-carboxamide** **(BT-308)**. A yellow solid (95 mg, 44%). ^1^H NMR (CDCl_3_, 600 MHz) δ 10.56 (s, 1H), 8.86 (s, 1H), 8.17 (d, J = 2.4 Hz, 1H), 7.86 (d, *J* = 9.0, 1H), 7.82 (m, 2H), 7.63 (d, *J* = 8.4 Hz, 1H), 7.23 (d, J = 9 Hz, 1H), 1.35 (s, 12H) ppm; ^13^C NMR (CDCl_3_, 150 MHz) δ 160.9, 159.03, 146.1, 141.8, 137.7, 134.1, 133.8, 129.5, 129.0, 129.3, 123.9, 123.4, 121.8, 120.5, 118.7, 117.2, 85.9, 21.5 ppm. HRMS (EI) Calcd. for C_22_H_20_BClN_2_O_7_ [M+H]^+^ requires 471.1130, found 471.1273.

**6-chloro-2-oxo-N-(4-(trifluoro-l4-boraneyl)phenyl)-2H-chromene-3-carboxamide, potassium salt** **(BT-323)**. A yellow solid (38 mg, 95%). ^1^H NMR (CDCl_3_, 300 MHz) δ 10.42 (s, 1H), 8.86 (s, 1H), 8.14 (s, 1H), 7.78 (d, J = 9.0 Hz, 1H), 7.58 (d, J = 9.0 Hz, 1H), 7.41 (d, *J* = 7.8 Hz, 2H), 7.3 (d, J = 7.8 Hz); ^13^C NMR (CDCl_3_, 75 MHz) δ 160.7, 159.3, 152.9, 146.2, 135.5, 133.9, 132.3, 129.5, 129.3, 121.8, 120.4, 118.7, 118.6, 118.35. HRMS (EI) Calcd. for C_16_H_9_BClF_3_KNO_3_ [M+H]^+^ requires 427.9851, found 427.1125.

**6-chloro-N-(4-hydroxyphenyl)-2-oxo-2H-chromene-3-carboxamide** **(BT-317)**. A yellow solid (90 mg, 61%). ^1^H NMR (CDCl_3_, 300 MHz) δ 10.4 (s, 1H), 9.37 (s, 1H), 8.83 (s, 1H), 8.13 (d, J = 2.4 Hz, 1H), 7.79 (dd, *J_1,2_* = 9.0, 2.4 Hz, 1H), 7.57 (d, J = 8.4 Hz, 1H), 7.49 (d, *J* = 8.4 Hz, 2H), 6.75 (d, J = 9 Hz, 3H) ppm; ^13^C NMR (CDCl_3_, 75 MHz) δ 160.7, 160.0, 152.5, 146.1, 135.3, 134.7, 133.4, 131.5, 129.0, 128.8, 125.6, 120.4, 119.9, 118.2. HRMS (EI) Calcd. for C_16_H_10_ClNO_4_ [M]^+^ requires 338.0196, found 338.1766.

**6-chloro-2-oxo-N-(4-(trifluoro-l4-boraneyl)benzyl)-2H-chromene-3-carboxamide, potassium salt (BT-318)**. A yelow solid (71 mg, 68%). ^1^H NMR (CDCl_3_, 300 MHz) δ 8.92 (t, J = 5.4 Hz, 1H), 8.82 (s, 1H), 8.11 (d, *J* = 2.4 Hz, 1H), 7.75 (dd, *J_1,2_* = 9.0, 2.4 Hz, 1H), 7.53 (d, *J* = 8.4 Hz, 1H), 7.26 (d, J = 7.8 Hz, 2H), 7.05 (d, *J*  = 7.2 Hz, 2H), 4.43 (d, *J* = 6.0 Hz, 2H); ^13^C NMR (CDCl_3_, 75MHz) δ 161.2, 160.4, 152.9, 146.5, 135.7, 135.1, 133.1, 131.9, 129.2, 129.2, 120.8, 120.3, 118.6, 43.6 ppm. HRMS (EI) Calcd. for C_17_H_11_BClF_3_KNO_3_ [M+H]^+^ requires 420.0188, found 420.1199.

**General procedure for synthesis of 2H-chromene ester derivative:**


 The 6-chloro-2-oxo-2H-chromene-3-carboxylic acid (2.0 mmol) was suspended in oxalyl chloride (3 equiv) for 12 hrs at room temperature. The resulting solution was evaporated to dryness and the residue was dispersed in dry toluene (10 mL). The reaction temperature was cooled to 0 ^o^C in an ice bath and triethyl amine (3 equiv) and 4-hydroxy phenyl boronic acid pinacol ester (1.1 equiv) was added slowly, and then the reaction mixture was stirred at room temperature for 12 hrs. After completion of the reaction, aqueous sodium hydroxide was added and then extracted with DCM (10 × 3 mL). The combined organic layers were dried over Na_2_SO_4_ and filtered. The solvent was removed *in vacuo* and the residue was purified by a silica gel chromatography to afford the expected product.

**4-(4,4,5,5-tetramethyl-1,3,2-dioxaborolan-2-yl)phenyl 6-chloro-2-oxo-2H-chromene-3-carboxylate (BT-306)**. A brown solid (300 mg, 71%). ^1^H NMR (DMSO, 600 MHz): δ 9.02 (s, 1H), 8.10 (d, J = 2.4 Hz, 1H), 7.81 (dd, *J_1,2_* = 8.9, 2.6 Hz, 1H), 7.76 (d, *J* = 8.3 Hz, 2H), 7.52 (d, J = 8.9 Hz, 1H), 7.28 (d, *J* = 8.3 Hz, 2H), 1.29 (s, 12H) ppm; ^13^C NMR (DMSO, 150 MHz) δ 161.0, 155.8, 153.9, 153.2, 150.2, 149.1, 136.8, 136.5, 129.8, 129.0, 121.8, 119.6, 118.8, 117.9, 84.3, 25.1 ppm; HRMS (EI) Calcd. for C_22_H_20_BClO_6_ [M+H]^+^ requires 427.1120, found 427.1125.

**4-(trifluoro-l4-boraneyl)phenyl 6-chloro-2-oxo-2H-chromene-3-carboxylate, potassium salt** **(BT-321)**. A brown solid (58 mg, 57%). ^1^H NMR (DMSO, 600 MHz): δ 8.98 (s, 1H), 8.09 (d, J = 3.0 Hz, 1H), 7.79 (dd, *J1,2* = 9.0, 2.4 Hz, 1H), 7.51 (d, *J* = 9.0 Hz, 1H), 7.37 (d, J = 8.4 Hz, 2H), 6.93 (d, *J* = 8.4 Hz, 2H); ^13^C NMR (CDCl_3_, 75 MHz) δ 161.6, 156.0, 153.8, 149.0,148.6, 136.0, 134.6, 132.7, 129.7, 128.9, 119.6, 118.7, 118.6. HRMS (EI) Calcd. for C_16_H_8_BClF_3_KNO_4_ [M+H]^+^ requires 427.9851, found 427.8827.

**General Precedures for the synthesis of oxadiazole compound**.

The appropriate coumarinic acid (0.5 mmol) and CDI (carbonyldiimidazole, 0.5 mmol) in dry DMF (2 mL) was stirred at 80 ^o^C for 20 mins. The corresponding amidoxime derivative (1.1 equiv) was added to the resultant turbid solution. This solution was stirred at 110 ^o^C for 12 hrs. Then the precipitate formed was isolated through filtration and washed with isopropanol. This crude solid was purified by silica gel chromatography to afford the product.

**3-(3-(3-bromo-4-methoxyphenyl)-1,2,4-oxadiazol-5-yl)-6-chloro-2H-chromen-2-one (BT-315)**. A yellow solid (65 mg, 30%). ^1^H NMR (CDCl_3_, 300 MHz) δ 8.03 (s, 1H), 7.87 (d, *J* = 7.8 Hz, 2H), 7.63 (d, *J* = 8.4 Hz, 3H), 6.99 (s, 1H), 6.57-6.52 (m, *J* = 8.4 Hz, 2H); ^13^C NMR (CDCl_3_, 75 MHz) δ 160.8, 1591, 152.8,146.1,137.7, 134.0, 133.8, 130.8, 130.2, 129.4, 129.1, 128.9, 127.6, 124.0, 123.5, 121.7, 120.4, 118.7, 117.4, 116.7 ppm. HRMS (EI) Calcd. for C_18_H_10_BrClN_2_O_4_ [M+H]^+^ requires 431.9512, found 431.9183.

**3-(3-(4-bromophenyl)-1,2,4-oxadiazol-5-yl)-6-chloro-2H-chromen-2-one (BT-322)**. A yellow solid (100 mg, 49%). m.p. 136-138 ^o^C. ^1^H NMR (CDCl_3_, 300 MHz) δ 9.12 (s, 1H), 8.16 (s, 1H), 8.01 (d, *J* = 7.8 Hz, 2H), 7.83 (d, *J* = 8.4 Hz, 3H), 7.57 (d, *J* = 8.4 Hz, 1H); ^13^C NMR (CDCl_3_, 75 MHz) δ 172.7, 167.6, 146.9, 143.8, 133.5, 133.1, 132.3, 130.0, 129.0, 125.4, 119.8, 113.6 ppm. HRMS (EI) Calcd. for C_17_H_8_BrClN_2_O_3_ [M+H]^+^ requires 404.6240, found 404.9426.

**General Precedure for the synthesis of chromene aminonitrile Coumpound**

Into a 10-mL round bottomed flask were added chromene aldehyde derivative (1.0 mmol), amine (1.0 mmol), TMSCN (1.2 mmol), H_2_O (2 mL), and InCl_3_ (0.1 mmol) sequentially. The reaction mixture was stirred vigorously at room temperature and the progress of the reaction was monitored by TLC. After stirring for 4–6 h at room temperature the solid that was formed was filtered and washed with water and hexane to yield the desired product.


**2-(6,8-dichloro-2-phenyl-2H-chromen-3-yl)-2-((4-methyl-3-(4,4,5,5-tetramethyl-1,3,2-dioxaborolan-2-yl)phenyl)amino)acetonitrile (BT-395)**. A yellow solid (185 mg, 34%). ^1^H NMR (CDCl_3_, 300 MHz) δ ^1^H NMR (CDCl_3_, 300 MHz) δ 9.72 (s, 1H), 7.30-7.26 (m, 10H), 7.15 (m, 2H), 6.46 (s, 1H), 3.52 (s, 1H), 1.33 (s, 12H); ^13^C NMR (CDCl_3_, 75 MHz)  159.0, 148.1, 136.0, 135.6, 132.5, 132.1, 130.7, 129.1, 128.9, 128.2, 127.3, 127.2, 126.9, 125.9, 124.7, 123.3, 122.1, 121.0, 120.5, 84.1, 75.5, 25.4 ppm. HRMS (EI) Calcd. for C_30_H_29_BCl_2_N_2_O_3_ [M+H]^+^ requires 547.2830, found 547.3381.

**2-(6,8-dichloro-2-phenyl-2H-chromen-3-yl)-2-((4-methyl-3-(trifluoro-l4-boraneyl)phenyl)amino)acetonitrile, potassium salt (BT-397)**. A yellow solid (185 mg, 34%). ^1^H NMR (CDCl_3_, 300 MHz) δ ^1^H NMR (CDCl_3_, 300 MHz) δ 9.72 (s, 1H), 7.30-7.26 (m, 10H), 7.15 (m, 2H), 6.46 (s, 1H), 3.52 (s, 1H), 1.33 (s, 12H); ^13^C NMR (CDCl_3_, 75 MHz) δ 159.0, 148.1, 136.0, 135.6, 132.5, 132.1, 130.7, 129.1, 128.9, 128.2, 127.3, 127.2, 126.9, 125.9, 124.7, 123.3, 122.1, 121.0, 120.5, 84.1, 75.5, 25.4 ppm. HRMS (EI) Calcd. for C_30_H_29_BCl_2_N_2_O_3_ [M+H]^+^ requires 547.2830, found 547.3381.

**Synthesis of BT-399**

**(E)-1-phenyl-N-(3-(4,4,5,5-tetramethyl-1,3,2-dioxaborolan-2-yl)phenyl)methanimine (3):** A oven dried round bottom flask (RBF) was charged with amine **1** (1 equiv.) and aldehyde **2** (1 equiv.) in methanol. The reaction mixture was stirred at room temperature for 12 h. Progress of the reaction was monitored by TLC. After completion of the reaction, methanol was evaporated on rota vapor, white solid product was observed. Which was further purified by recrystallization (hot ethanol).

**N-benzyl-3-(4,4,5,5-tetramethyl-1,3,2-dioxaborolan-2-yl) aniline (4):**

An oven dried round bottom flask (RBF) was charged with imine **1** (1 equiv.) and dissolved in methanol. To the reaction solution, NABH_4_ (1 equiv.) was added slowly at 0 °C. The resulted reaction mixture was stirred at room temperature for 12 h. After completion of the reaction, methanol was evaporated on rota vapor, further NABH_4_ was neutralized by 1N, HCl solution. After neutralization, ethyl acetate was added in the reaction mixture; the combined organic layer was collected and dried with Na_2_SO_4_ and evaporated under reduced pressure. The crude material was purified by column chromatography (5% ethyl acetate: 95 % hexane).

**N-benzyl-2-chloro-N-(3-(4,4,5,5-tetramethyl-1,3,2-dioxaborolan-2-yl) phenyl) acetamide (BT-399):** An oven dried round bottom flask (RBF) was charged with **4** (1 equiv.) and dissolved in DMF. To the reaction solution, chloroacetyl chloride (1 equiv.) was added slowly at 0 °C. The resulted reaction mixture was stirred at room temperature for 12 h. After completion of the reaction, ethyl acetate was added in the reaction mixture; the combined organic layer was collected and dried with Na_2_SO_4_ and evaporated under reduced pressure. The crude material was purified by column chromatography (10% ethyl acetate: 90 % hexane).

White solid ( Yield = 75 %),^1^H NMR (500 MHz, CDCl_3_) δ 7.77 (d, *J* = 7.4 Hz, 1H), 7.53 (d, *J* = 0.8 Hz, 1H), 7.31 (t, *J* = 7.6 Hz, 1H), 7.07 (s, 4H), 6.97 (d, *J* = 7.9 Hz, 1H), 4.86 (s, 2H), 3.83 (s, 2H), 2.31 (s, 3H), 1.35 (s, 12H). ^13^C NMR (125 MHz, CDCl_3_) δ 165.94, 140.33, 137.25, 134.90, 134.01, 133.49, 131.38, 129.10, 129.07, 129.02, 84.22, 53.46, 42.36, 24.85, 21.14. HRMS (ESI) calcd for C_22_H_28_BClNO_3_ 400.1845 found 400.1867 [M+H]^+^.
